# A yeast platform for high-level synthesis of tetrahydroisoquinoline alkaloids

**DOI:** 10.1038/s41467-020-17172-x

**Published:** 2020-07-03

**Authors:** Michael E. Pyne, Kaspar Kevvai, Parbir S. Grewal, Lauren Narcross, Brian Choi, Leanne Bourgeois, John E. Dueber, Vincent J. J. Martin

**Affiliations:** 1 Department of Biology, Concordia University, Montréal, QC Canada; 2 Centre for Applied Synthetic Biology, Concordia University, Montréal, QC Canada; 3 Department of Chemical and Biomolecular Engineering, University of California, Berkeley, Berkeley, CA USA; 4 Department of Bioengineering, University of California, Berkeley, Berkeley, CA USA; 5 Biological Systems & Engineering Division, Lawrence Berkeley National Laboratory, Berkeley, CA USA

**Keywords:** Metabolic engineering, Natural product synthesis, Applied microbiology

## Abstract

The tetrahydroisoquinoline (THIQ) moiety is a privileged substructure of many bioactive natural products and semi-synthetic analogs. Plants manufacture more than 3,000 THIQ alkaloids, including the opioids morphine and codeine. While microbial species have been engineered to synthesize a few compounds from the benzylisoquinoline alkaloid (BIA) family of THIQs, low product titers impede industrial viability and limit access to the full chemical space. Here we report a yeast THIQ platform by increasing production of the central BIA intermediate (*S*)-reticuline to 4.6 g L^−1^, a 57,000-fold improvement over our first-generation strain. We show that gains in BIA output coincide with the formation of several substituted THIQs derived from amino acid catabolism. We use these insights to repurpose the Ehrlich pathway and synthesize an array of THIQ structures. This work provides a blueprint for building diverse alkaloid scaffolds and enables the targeted overproduction of thousands of THIQ products, including natural and semi-synthetic opioids.

## Abstract (web summary)

Plants synthesize more than 3000 tetrahydroisoquinoline (THIQ) alkaloids, but only a few of them have been produced by engineered microbes and titers are very low. Here, the authors increase (*S*)-reticuline titer to 4.6 g/L and repurpose the yeast Ehrlich pathway to synthesize a diverse array of THIQ scaffolds.

## Introduction

Specialized metabolites equip plants with a chemical framework for communication and defense, while many such natural products have been exploited for use as flavors, dyes, and pharmaceuticals^1^. Crop-based manufacturing has enabled production of some plant metabolites at commercial scale^2^, yet most high-value products are synthesized at very low concentrations by native producers. Although some natural products can be attained through chemical synthesis, the structural complexity of plant metabolites and a lack of stereocontrol result in unfeasible synthetic routes. Microbial biosynthesis has potential to overcome many of these hurdles and holds promise as a viable alternative to traditional modes of chemical and pharmaceutical manufacturing. Landmark successes in this arena, such as industrial-scale production of the anti-malarial precursor artemisinic acid^3^ and the chemical building block β-farnesene^4^, have paved the way for new opportunities in microbial biomanufacturing.

The tetrahydroisoquinoline (THIQ) structural moiety forms the basis of >3000 plant natural products^5^, as well as a suite of synthetic and semi-synthetic pharmaceuticals. Naturally occurring THIQ metabolites include the benzylisoquinoline, phenethylisoquinoline, ipecac, and Amaryllidaceae alkaloid classes^5^. The peyote cactus produces a number of simple substituted and unsubstituted THIQs^6^, while species of *Erythrina* synthesize complex spiro-THIQ alkaloids^7^. Each of these metabolite classes possesses the privileged THIQ substructure that imparts a vast array of bioactivities following derivatization in downstream tailoring reactions. The Amaryllidaceae alkaloid galantamine and the phenethylisoquinoline colchicine are commercial THIQ-derived drugs used in the treatment of Alzheimer’s disease and gout, respectively^5^. Other synthetic and semi-synthetic THIQs approved by the FDA include the anti-Parkinson drug apomorphine, the chemotherapeutic trabectedin, the muscle relaxant cisatracurium, the anti-parasitic praziquantel, and the anti-chorea tetrabenazine used in the treatment of Huntington’s disease^8^.

The benzylisoquinoline alkaloids (BIAs) are the largest class of THIQ natural products and include several of the most important human medicines^9^. Morphine, codeine, and their analogs are potent BIA analgesics included in the World Health Organization’s List of Essential Medicines^10^. Papaverine is a vasodilator and antispasmodic drug, and noscapine exhibits promising anticancer properties^9^. Although global demand for morphinan BIAs is presently met through extraction from opium poppy (*Papaver somniferum*), most BIAs do not accumulate to sufficient concentrations in plant tissues. To begin exploring this untapped natural diversity, plant pathways mediating synthesis of noscapine, sanguinarine, morphine, codeine, and hydrocodone have been reconstructed in yeast^11–14^. Despite these achievements, present yeast BIA titers have been limited to <2 mg L^−1^ (refs. ^15–18^). For instance, the hydrocodone pathway has been reconstituted within a single yeast strain at a titer of only 0.0003 mg L^−1^ (ref. ^16^) largely due to inefficiencies in formation of the dedicated THIQ precursor. Higher levels of the key intermediate (*S*)-reticuline have been reached in *E. coli* cultures (160 mg L^−1^)^19^, yet bacteria lack membrane-bound organelles required for functional expression of the numerous cytochrome P450 enzymes in downstream BIA pathways. Consequently, engineering *E. coli* for total biosynthesis of morphinan BIAs required partitioning the pathway amongst four engineered strains^20^. Scalable production of BIAs would be facilitated through the reconstruction of heterologous pathways in a single yeast strain^16,17^.

An emerging objective of synthetic biology is directed at expanding natural product diversity by engineering structural scaffolds and chemical modifications that are not observed in nature^21^. Although many biosynthetic enzymes exhibit broad substrate specificities when assayed in vitro, natural pathways have evolved a preference for a single substrate or very small subset of accepted building blocks^22^. The Pictet-Spengler condensation between an aryl amine and a carbonyl compound underlies the synthesis of >3000 THIQ alkaloids^5^, yet much of this diversity arises from only four aldehyde species (4-hydroxyphenylacetaldehyde, 4-hydroxydihydrocinnamaldehyde, protocatechuic aldehyde, and secologanin). As Pictet-Spenglerases are able to accept a tremendous range of carbonyl substrates^7,23–26^, natural THIQ alkaloids occupy a miniscule fraction of the conceivable chemical space. To access this untapped potential, norcoclaurine synthase (NCS) has been exploited for the synthesis of novel substituted THIQs^24,26^, including ones derived from ketone building blocks^7^. These in vitro approaches involve the synthesis of complex and unusual carbonyl substrates; consequently, producing such compounds at scale still presents a formidable challenge.

Here, we establish an efficient biosynthetic route to THIQ alkaloids in which diverse structures are synthesized in vivo from simple substrates. We target the BIA family of THIQs for overproduction by increasing yeast output of (*S*)-reticuline to 4.6 g L^−1^, exemplifying a 57,000-fold improvement over our previous work^15^. By inactivating a functionally redundant subset of seven host oxidoreductases (Ari1, Adh6, Ypr1, Ydr541c, Aad3, Gre2, and Hfd1), we solve a long-standing challenge associated with yeast fusel metabolism^27^, as *S. cerevisiae* rapidly transforms a key BIA pathway precursor (4-hydroxyphenylacetaldehyde) to its corresponding acid or alcohol. As our THIQ platform accumulates both aromatic and aliphatic aldehydes, we further showcase its biosynthetic potential by synthesizing >10 diverse THIQ scaffolds from endogenous and fed amino acids. Altogether, these efforts expand the diversity of privileged THIQ structures beyond natural products and outline a general synthetic biology framework for building chemical scaffolds in yeast.

## Results

### A yeast platform for synthesis of tetrahydroisoquinolines

**Fig. 1:**
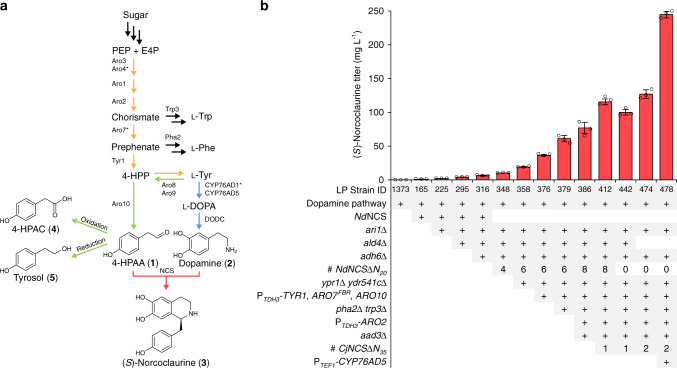
Engineering a THIQ-producing yeast. **a** (*S*)-Norcoclaurine (**3**) synthetic pathway in engineered yeast. The native yeast Ehrlich pathway (green) supplies 4-HPAA (**1**) from l-tyrosine, while a heterologous pathway (blue) generates dopamine (**2**), also from l-tyrosine. CYP76AD5 is a more active plant tyrosine hydroxylase compared to CYP76AD1* (CYP76AD1^W13L F309L^), which is an engineered variant of CYP76AD1. NCS catalyzes an enantioselective Pictet-Spengler condensation of 4-HPAA and dopamine, yielding (*S*)-norcoclaurine (pink). Native shikimate and l-tyrosine biosynthetic pathways are shown in orange. **b** (*S*)-Norcoclaurine (**3**) titer in culture supernatants of successive engineered strains. *Cj*NCS stereo-specifically yields (*S*)-norcoclaurine^64^, while the stereo-specificity of *Nd*NCS has not been reported. Error bars represent the mean ± s.d. of *n* = 3 independent biological samples. All strains exhibited a significant increase (*P* < 0.05) in (*S*)-norcoclaurine titer relative to the respective parental strain, with the exception of strain LP442, which exhibited a significant decrease (*P* < 0.05) in titer relative to strain LP412. Statistical differences between control and derivative strains were tested using two-tailed Student’s *t*-test. Refer to Supplementary Data 3 for genotypes of the full 23-strain (*S*)-norcoclaurine lineage. l-DOPA l-3,4-dihydroxyphenylalanine, DODC DOPA decarboxylase, E4P erythrose-4-phosphate, 4-HPAA 4-hydroxyphenylacetaldehyde, 4-HPAC 4-hydroxyphenylacetic acid, 4-HPP 4-hydroxyphenylpyruvate, NCS norcoclaurine synthase, PEP phosphoenolpyruvate, l-Phe l-phenylalanine, l-Trp l-tryptophan, l-Tyr l-tyrosine. Source data underlying Fig. 1b are provided in a Source Data file.

To improve biosynthesis of THIQs in yeast, we focused on the first committed reaction and major rate-limiting step of the canonical BIA pathway. This conversion involves condensation of 4-hydroxyphenylacetaldehyde (4-HPAA, **1**) and dopamine (**2**) by NCS, yielding (*S*)-norcoclaurine (**3**) (Fig. 1a). We improved production of (*S*)-norcoclaurine >11,000-fold compared to our previous efforts^15^ by implementing >20 successive strain modifications to the yeast shikimate, Ehrlich, and l-tyrosine metabolic pathways (Fig. 1b; strain LP478). (*S*)-Norcoclaurine synthesis in our early strains was limited by 4-HPAA, which is rapidly transformed to the corresponding fusel acid (4-hydroxyphenylacetic acid; 4-HPAC, **4**) or alcohol (tyrosol, **5**) via the Ehrlich pathway. The specific consortium of enzymes mediating these redox reactions has thus far evaded identification^27^, as yeast produces a suite of >30 oxidoreductases^28^. A gene deletion screen of >20 of these candidates implicated the Ari1 short-chain dehydrogenase/reductase in 4-HPAA reduction (Supplementary Fig. 1). Owing to substantial functional redundancy in yeast oxidoreductases, we proceeded to delete a total of five genes encoding NADPH-dependent reductases and dehydrogenases (in order: *ari1*Δ *adh6*Δ *ypr1*Δ *ydr541c*Δ *aad3*Δ; Supplementary Fig. 2), enabling the production of 77 mg L^−1^ of (*S*)-norcoclaurine by strain LP386 (Fig. 1b). Inactivation of redundant 4-HPAA reductases did not result in a decline in strain fitness (Supplementary Fig. 3; strain LP165 versus LP358), while strain LP478 exhibited a 38% decrease in maximum specific growth rate relative to the BY4741 parent strain in microtiter plate cultivations (0.32 h^−1^ and 0.20 h^−1^, respectively).

Although the *ALD4* aldehyde-dehydrogenase-encoding gene was deleted in early strains, resulting in gains in both dopamine and (*S*)-norcoclaurine titer (Supplementary Figs. 1 and 2), we reintroduced the gene in later strains (Fig. 1b; strains LP474 and LP478) to promote ethanol consumption in fed-batch fermentor cultures. To further improve precursor supply, strain LP478 incorporates overexpression of genes encoding chorismate synthase (*ARO2*), prephenate dehydrogenase (*TYR1*), and phenylpyruvate decarboxylase (*ARO10*), as well as feedback-resistant forms of 3-deoxy-d-arabino-heptulosonate-7-phosphate synthase (*ARO4*^*FBR*^) and chorismate mutase (*ARO7*^*FBR*^). We also inactivated the l-phenylalanine and l-tryptophan biosynthetic pathways through deletion of prephenate dehydratase (*pha2*Δ) and indole-3-glycerol-phosphate synthase (*trp3*Δ) genes, respectively.

Our initial (*S*)-norcoclaurine-producing strains contained an N-terminally truncated NCS variant from *Nandina domestica* (*Nd*NCSΔN_20_)^29^ (Supplementary Fig. 4). As (*S*)-norcoclaurine production increased by supplying additional copies of the *NdNCSΔN*_*20*_ gene (up to eight copies) (Fig. 1b and Supplementary Fig. 2), we set out to identify a more efficient NCS ortholog. Several groups have reported the use of truncation variants of NCS from *Coptis japonica* (*Cj*NCS)^17,24^. We explored extensive N-terminal truncations of *Cj*NCS by reducing the protein to the core Bet v1 domain and comparing NCS activity to *Nd*NCSΔN_20_. A 35 amino acid deletion (*Cj*NCSΔN_35_) facilitated the production of five-fold more (*S*)-norcoclaurine than an isogenic strain harboring *NdNCSΔN*_*20*_ (Supplementary Fig. 5). Implementation of *Cj*NCSΔN_35_ in strain LP386 possessing eight copies of *NdNCSΔN*_*20*_ yielded a 50% increase in (*S*)-norcoclaurine titer (strain LP412). To limit overproduction of heterologous proteins, we proceeded to delete all eight copies of *NdNCSΔN*_*20*_ (strain LP442) and integrated an additional copy of *CjNCSΔN*_*35*_ (strain LP474).

Concurrent precursor feeding experiments using strain LP412 revealed a greater increase in (*S*)-norcoclaurine production upon supplementation of l-DOPA compared to l-tyrosine (Supplementary Fig. 6), indicating a limitation in dopamine supply. To address this substrate imbalance, we introduced a superior tyrosine hydroxylase ortholog (CYP76AD5)^30–33^ into strain LP474, which already expressed an engineered CYP76AD1 variant (CYP76AD1^W13L F309L^)^15^ (Supplementary Fig. 7; strain LP478). In line with substrate supplementation experiments, increased expression of tyrosine hydroxylase through implementation of *CYP76AD5* doubled (*S*)-norcoclaurine titer (245 mg L^−1^) relative to strain LP474. Cultivation of strain LP478 in a pulsed sucrose fed-batch fermentor yielded 1.6 g L^−1^ of (*S*)-norcoclaurine (Supplementary Fig. 8). Thus, upregulating the yeast l-tyrosine and Ehrlich pathways and stabilizing a 4-HPAA precursor supply enabled increased titers of BIAs that are synthesized directly from sugar using a fed-batch process. Chiral analysis of a late-stage fed-batch fermentor sample confirmed that all of the norcoclaurine generated by strain LP478 was the (*S*)-enantiomer (Supplementary Fig. 8).

### Production of functionalized substituted THIQs

**Fig. 2:**
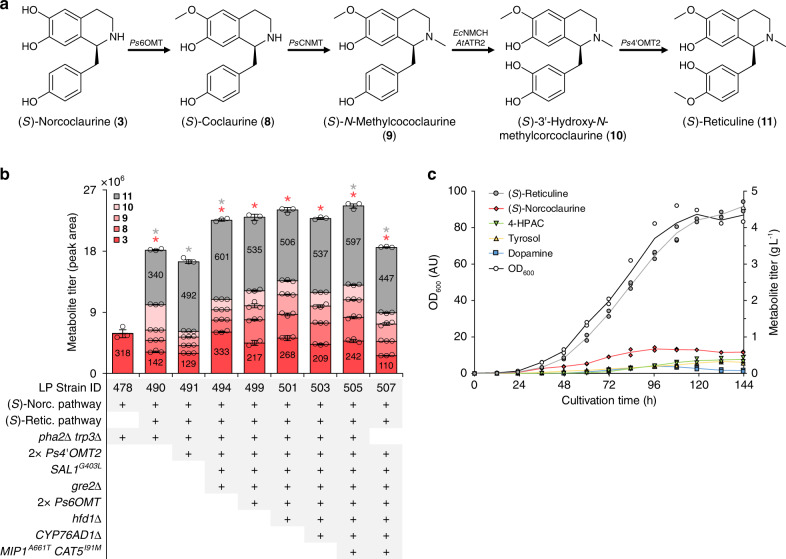
Extending the canonical THIQ pathway to (*S*)-reticuline. **a** Composite pathway for synthesis of (*S*)-reticuline (**11**) from (*S*)-norcoclaurine (**3**). **b** Relative titers of (*S*)-reticuline pathway intermediates in culture supernatants of successive engineered production strains. Peak area counts are shown for all pathway intermediates and absolute titers are depicted in mg L^–1^ for (*S*)-norcoclaurine and (*S*)-reticuline. Error bars represent the mean ± s.d. of *n* = 3 independent biological samples. Asterisk (*) denotes a significant increase or decrease (*P* < 0.05) in (*S*)-norcoclaurine or (*S*)-reticuline titer relative to the parental strain. Statistical differences between control and derivative strains were tested using two-tailed Student’s *t*-test. **c** Cultivation of an (*S*)-reticuline-producing strain (LP507 harboring pHUM) in a sucrose-pulsed fed-batch fermentor using a simple mineral medium. Growth of biomass (OD_600_) and accumulation of BIA metabolites in the culture medium were measured during cultivation. Data points from duplicate experiments are shown and the mean is depicted as a line. Source data underlying Fig. 2b, c are provided in a Source Data file.

Having achieved >1 g L^−1^ of (*S*)-norcoclaurine, we sought to extend the BIA pathway to the major branch point intermediate (*S*)-reticuline. This four-step conversion involves methylation of the THIQ motif of (*S*)-norcoclaurine (**3**) by two methyltransferases from opium poppy (*Papaver somniferum*; *Ps*6OMT and *Ps*CNMT), yielding (*S*)-coclaurine (**8**) followed by (*S*)-*N*-methylcoclaurine (**9**) (Fig. 2a). The benzyl moiety of (*S*)-*N*-methylcoclaurine is then hydroxylated by a cytochrome P450 *N*-methylcoclaurine hydroxylase from California poppy (*Eschscholzia californica*; CYP80B1 or *Ec*NMCH)^15^, yielding (*S*)-3ʹ-hydroxy-*N*-methylcoclaurine (**10**), which is subsequently methylated by a 4ʹ*O*-methyltransferase (*Ps*4ʹOMT2) to generate (*S*)-reticuline (**11**). Our (*S*)-reticuline pathway module included a cytochrome P450 reductase (CPR) from *Arabidopsis thaliana* (*At*ATR2) for electron transfer to *Ec*NMCH. Introduction of this composite pathway to strain LP478 generated 340 mg L^−1^ of (*S*)-reticuline in microtiter plate cultivations (Fig. 2b; strain LP490). All three intermediates in the conversion of (*S*)-norcoclaurine (**3**) to (*S*)-reticuline (**11**) were detected. Assuming an equal ionization efficiency, (*S*)-3ʹ-hydroxy-*N*-methylcoclaurine (**10**) was found to accumulate to the greatest extent. To address this bottleneck, we integrated an additional copy of *Ps4*ʹ*OMT2* (strain LP491), which nearly abolished accumulation of (*S*)-3ʹ-hydroxy-*N*-methylcoclaurine and resulted in a 45% improvement in (*S*)-reticuline titer to 492 mg L^−1^.

We measured fusel products generated by strain LP491 in microtiter plate cultures and observed substantial accumulation of tyrosol (235 mg L^−1^) despite deletion of five oxidoreductase genes, implying that additional unidentified enzymes reduce 4-HPAA to tyrosol. Single-gene deletions of seven of these candidates (*AAD4*, *AAD14*, *ADH7*, *GCY1*, *GRE2*, *SFA1*, and *YGL039W*) in strain LP491 identified Gre2 as a potent 4-HPAA reductase (Supplementary Fig. 9), as its inactivation facilitated an 83% decrease in tyrosol formation in microtiter plate cultures (Supplementary Fig. 10; strain LP494). In line with this reduction, (*S*)-reticuline and (*S*)-norcoclaurine titers increased by 22% and 158%, respectively (Fig. 2b). Growth of strain LP494 in a pulsed fed-batch fermentor produced only 138 mg L^−1^ of tyrosol, whereas the (*S*)-norcoclaurine-producing strain LP478 cultivated under identical conditions generated 2.1 g L^−1^ of tyrosol (Supplementary Fig. 8), corresponding to a 93% reduction in tyrosol synthesis and improved flux to the heterologous pathway (Supplementary Fig. 10).

With tyrosol production nearly abolished, 4-HPAC increased in concentration and became the dominant fusel product in both microtiter plate and fed-batch fermentor cultures (Supplementary Fig. 10). We thus set out to identify the enzyme(s) responsible for the oxidation of 4-HPAA to 4-HPAC. We surveyed yeast aldehyde dehydrogenases for cytosolic enzymes that exhibit activity on aromatic aldehydes, which directed our attention to Hfd1, a dual-function aldehyde-dehydrogenase involved in ubiquinone (coenzyme Q_10_) biosynthesis and fatty acid catabolism. One of the physiological substrates of Hfd1 is 4-hydroxybenzaldehyde^34^, a close structural analog of 4-HPAA, and localization studies have shown that Hfd1 resides in the outer mitochondrial membrane where it is ostensibly exposed to the cytosol^35^. Deletion of *HFD1* in our LP494 *gre2*Δ mutant strain (yielding strain LP498) decreased 4-HPAC production by >80% in both microtiter plate and pulsed fed-batch fermentor cultures (Supplementary Fig. 11). Strain LP501, which incorporates *gre2*Δ and *hfd1*Δ mutations, produced 3.1 g L^−1^ of (*S*)-reticuline and 0.7 g L^−1^ of (*S*)-norcoclaurine in a pulsed fed-batch fermentor (Supplementary Fig. 12). Tyrosol, 4-HPAC, and dopamine reached final concentrations of <0.3 g L^−1^ each.

Having efficiently redirected carbon from fusel product synthesis to BIA production, we sought to improve strain fitness under fed-batch conditions, as greater product titers are achieved at higher cell densities. We first deleted the gene encoding CYP76AD1^W13L F309L^ (strain LP503) owing to its non-productive side reaction and reduced catalytic efficiency relative to CYP76AD5^33^. We also repaired three well-known mutations harbored by S288C-derived strains that together destabilize the mitochondrial genome (*SAL1*^*G403L*^, *MIP1*^*A661T*^, *CAT5*^*I91M*^; strain LP505)^36^. Finally, we restored l-tryptophan and l-phenylalanine biosynthesis pathways via reintroduction of *TRP3* and *PHA2*, respectively (strain LP507), which decreases product concentrations in microtiter plates (Fig. 2b), yet eliminates the requirement for exogenous supplementation of l-tryptophan and l-phenylalanine. Strain LP507 produced 4.6 g L^−1^ of (*S*)-reticuline and 0.6 g L^−1^ of (*S*)-norcoclaurine in a pulsed fed-batch cultivation using a simple mineral medium (Fig. 2c).

### De novo synthesis of non-canonical tetrahydroisoquinolines

**Fig. 3:**
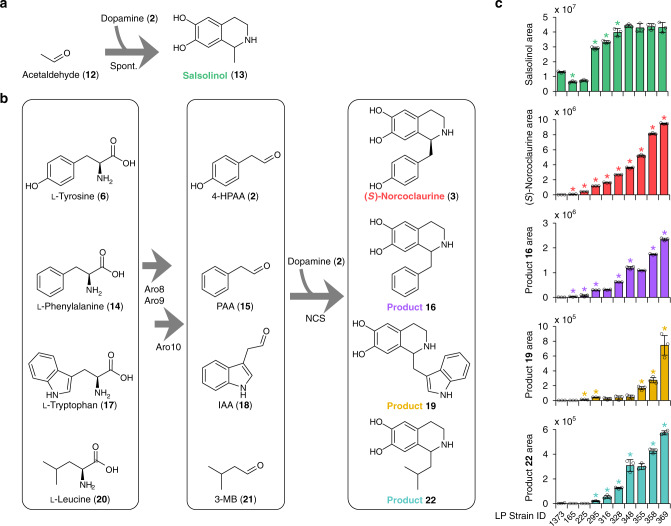
De novo synthesis of substituted tetrahydroisoquinolines in strains engineered for (*S*)-norcoclaurine production. **a** Formation of salsolinol (**13**) from acetaldehyde (**12**) and dopamine (**2**) occurs spontaneously in yeast strains engineered for dopamine production. **b** NCS-catalyzed formation of substituted THIQs through catabolism of endogenous amino acids via the Ehrlich pathway. Amino acids are converted to the respective aldehyde species via sequential transamination (Aro8/Aro9) and decarboxylation (Aro10) reactions. In the presence of dopamine (**2**) and NCS, aldehydes are converted to the corresponding substituted THIQs. Stereochemistry of non-canonical substituted THIQs is omitted. **c** Relative levels of substituted THIQs in yeast strains engineered for (*S*)-norcoclaurine (**3**) production. Strain 1373 possesses the dopamine pathway and lacks an NCS biosynthetic enzyme. Refer to Fig. 1b and Supplementary Data 3 for genotypes of engineered strains. All of the depicted substituted THIQs were synthesized de novo by strain LP295 and all subsequent engineered strains. Error bars represent the mean ± s.d. of *n* = 3 independent biological samples. Asterisk (*) denotes a significant increase or decrease (*P* < 0.05) in product titer relative to the precursor strain. Statistical differences between control and derivative strains were tested using two-tailed Student’s *t*-test. 4-HPAA 4-hydroxyphenylacetaldehyde, IAA indole acetaldehyde, 3-MB 3-methylbutanal, PAA phenylacetaldehyde, spont. spontaneous. Source data underlying Fig. 3c are provided in a Source Data file.

In addition to benzylisoquinolines, higher plants synthesize phenethylisoquinolines and the Amaryllidaceae alkaloids via 4-hydroxydihydrocinnamaldehyde and protocatechuic aldehyde, respectively. These natural pathways as well as in vitro studies^7,24,26,37^ highlight the broad substrate specificity of Pictet-Spenglerases and prompted us to employ our engineered strains to explore NCS promiscuity. We examined liquid chromatography–mass spectrometry (LC–MS) spectra derived from supernatants of an (*S*)-norcoclaurine production strain for peaks indicative of substituted THIQ products. Our search consisted of 64 theoretical THIQ products derived through Pictet-Spengler condensation of dopamine and endogenous yeast carbonyl species. From this search we identified four putative LC–MS peaks (Supplementary Data 1). A major peak corresponding to salsolinol (**13**), derived from condensation of dopamine (**2**) and acetaldehyde (**12**) (Fig. 3a), was observed in supernatants of all dopamine-producing strains irrespective of the presence of an NCS biosynthetic enzyme. Three additional LC–MS peaks were consistent with substituted THIQs derived from condensation of dopamine (**2**) and aldehydes from the Ehrlich pathway^27^. These substituted THIQs (**16**, **19**, and **22**) are presumed to arise from l-phenylalanine (**14**), l-tryptophan (**17**), and l-leucine (**20**) catabolism via the respective aldehydes phenylacetaldehyde (PAA, **15**), indole acetaldehyde (IAA, **18**), and 3-methylbutanal (3MB, **21**) (Fig. 3b). Analogous substituted THIQs derived from catabolism of l-isoleucine and l-valine were not observed since α-substituted aldehydes are not well tolerated by NCS^38^. Targeted fragmentation of substituted THIQ products yielded spectra consistent with the presumed metabolite identities^39^ (Supplementary Fig. 13). Further, cultivating strain LP385 harboring *Cj*NCSΔN_35_ on l-tyrosine (**6**), l-phenylalanine (**14**), l-tryptophan (**17**), or l-leucine (**20**) as a sole nitrogen source increased the area of LC–MS peaks corresponding to (*S*)-norcoclaurine (**3**) and products **16**, **19**, and **22**, respectively (Supplementary Fig. 14), providing additional evidence that these substituted THIQs arise from amino acid degradation. In agreement with these results, inactivation of l-phenylalanine (*pha2*Δ) and l-tryptophan (*trp3*Δ) biosynthesis pathways dramatically reduced levels of **16** and **19**, respectively (Supplementary Fig. 15).

We analyzed LC–MS spectra from supernatants of our oxidoreductase gene deletion strains to monitor levels of putative substituted THIQ products (**16**, **19**, and **22**) relative to improvements in (*S*)-norcoclaurine concentration (Fig. 3c). These peaks were not identified in supernatants of a dopamine-producing strain lacking an NCS enzyme (strain 1373) and, with the exception of **16**, were not detected in the *Nd*NCS strain used as the basis of our oxidoreductase gene deletions (strain LP165). Overall, formation of these presumed products closely paralleled (*S*)-norcoclaurine, in which biosynthesis of **16**, **19**, and **22** increased in accordance with (*S*)-norcoclaurine throughout the oxidoreductase gene deletion lineage. Synthesis of **16** and **19** increased through deletion of *ARI1* (strain LP225), whereas deletion of *YPR1* (strain LP355) or *ADH6* (strain LP316) increased production of **19** and **22**, respectively. Deletion of *YDR541C* (strain LP358) enhanced levels of all presumed THIQs derived from the Ehrlich pathway.

### Synthesis of tetrahydroisoquinolines from fed amino acids

**Fig. 4:**
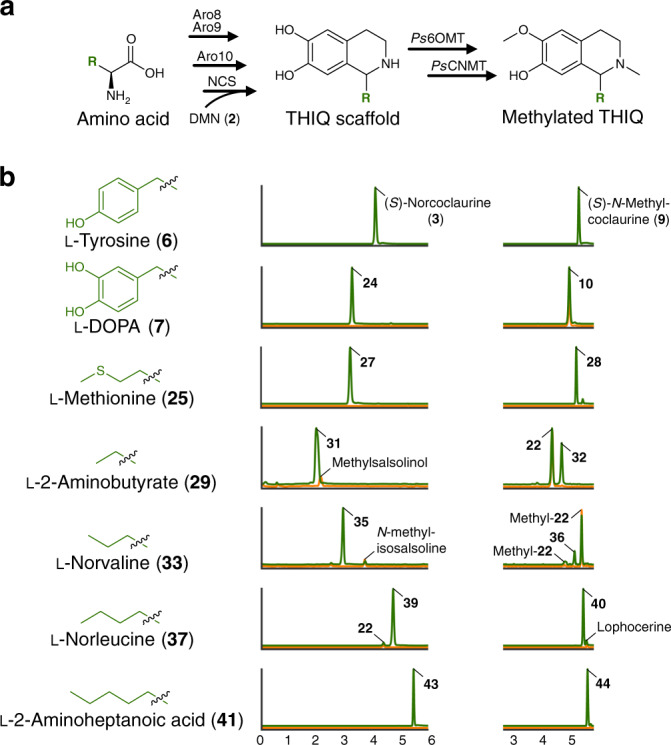
Synthesis and modification of substituted tetrahydroisoquinolines from supplemented amino acids. **a** NCS-catalyzed formation of substituted THIQs through catabolism of externally supplied amino acids via the Ehrlich pathway. **b** Ion-extracted LC–MS chromatograms of strain LP501 grown on l-methionine (**25**), l-DOPA (**7**), l-2-aminobutyrate (**29**), l-norvaline (**33**), l-norleucine (**37**), or l-2-aminoheptanoic acid (**41**) as the chief source of nitrogen (green). Aldehydes derived from amino acids were incorporated into the corresponding substituted THIQs and methylated by BIA tailoring enzymes (*Ps*6OMT and *Ps*CNMT) produced by strain LP501. Substituted THIQs and their methylated derivatives shifted in retention time relative to the canonical BIA products from l-tyrosine, namely (*S*)-norcoclaurine (**3**) and (*S*)-*N*-methylcoclaurine (**9**), which were formed de novo on all amino acid substrates. Growth of strain LP501 on urea as the major nitrogen source (orange) failed to generate peaks corresponding to substituted THIQs with the exception of **31** and **40**, which co-elute with singly methylated salsolinol (by *Ps*6OMT or *Ps*CNMT) and lophocerine, respectively. Product **36** elutes closely with both singly methylated derivatives of **22** (by *Ps*6OMT or *Ps*CNMT), which are synthesized de novo from l-leucine. Products **35** and **39** are isomers of *N*-methylisosalsoline and **22**, respectively, but do not co-elute with these de novo products. Methylation of norlaudanosoline (**24**) by *Ps*6OMT and *Ps*CNMT yields 3-hydroxy-*N*-methylcoclaurine (**10**), which is produced de novo by strain LP501 irrespective of the nitrogen source. All *m/z* values were calculated based on the expected structures of the respective compounds of interest (Supplementary Fig. 16 and Supplementary Table 1). DMN dopamine.

Owing to the capacity of our engineered host to accumulate aldehyde species and synthesize substituted THIQs from endogenous amino acids, we reasoned that supplying exogenous amino acids would enable the synthesis of additional THIQ structures. We devised a THIQ synthesis assay by cultivating an (*S*)-reticuline-producing strain (LP501; *ari1*Δ *adh6*Δ *ypr1*Δ *ydr541c*Δ *aad3*Δ *gre2*Δ *hfd1*Δ) on individual amino acids as the major source of nitrogen. In this manner, amino acid utilization via the Ehrlich pathway directly links cell growth to aldehyde and thus THIQ formation. Using this assay, we synthesized a diverse set of THIQs possessing both aliphatic and aromatic substitutions (Supplementary Fig. 16 and Supplementary Table 1). We first grew strain LP501 on l-DOPA, which is a non-proteinogenic derivative of l-tyrosine, suggesting that it serves as a substrate for enzymes of the Ehrlich pathway. Supplying l-DOPA (**7**) to strain LP501 as the major nitrogen source generated norlaudanosoline (tetrahydropapaveroline, **24**) via 3,4-dihydroxyphenylacetaldehyde (3,4-dHPAA, **23**). Norlaudanosoline (**24**) produced in this manner yielded a fragmentation spectrum identical to that of an authentic standard (Supplementary Fig. 17), thus validating our devised assay for synthesizing THIQ scaffolds. We extended this concept by feeding l-methionine (**25**) to strain LP501, which generated the presumed sulfur-containing THIQ **27** via methional (**26**) (Fig. 4). We also demonstrated production of ethyl-, propyl-, butyl-, and pentyl-substituted THIQs (**31**, **35**, **39**, and **43**, respectively) by supplying l-2-aminobutyrate (**29**), l-norvaline (**33**), l-norleucine (**37**), and l-2-aminoheptanoic acid (**41**), respectively (Fig. 4). Targeted fragmentation of substituted THIQ structures yielded spectra consistent with the presumed metabolite identities^39^ (Supplementary Fig. 17).

We also explored the promiscuity of BIA tailoring enzymes toward presumed substituted THIQs. Enzymes involved in methylation of (*S*)-norcoclaurine (*Ps*6OMT and *Ps*CNMT) exhibited activity on all substituted THIQs derived from supplemented amino acids (Fig. 4), as well as THIQ scaffolds synthesized de novo from endogenous substrates (Supplementary Fig. 18). Methylation of salsolinol (**13**) by *Ps*6OMT and *Ps*CNMT yielded *N*-methylisosalsoline (**45**), while methylation of the THIQ scaffold derived from l-leucine (**22**) gave rise to an LC–MS peak consistent with lophocerine (**48**), a naturally occurring THIQ alkaloid^40^. Thus, the pool of BIAs and other THIQs that can be synthesized using our high-flux THIQ-producing yeast is immensely diverse.

## Discussion

Our work demonstrates a major step towards industrial synthesis of microbially sourced THIQ pharmaceuticals by increasing production of the key BIA intermediate (*S*)-reticuline to 4.6 g L^−1^ using a simple mineral medium. Many bioprocesses become commercially viable when titers reach the gram per liter scale, yet very few complex plant natural products have been produced at this level by a microbial host^3,41,42^. A target of 5 g L^−1^ has been set forth for commercial-scale production of opioids^16^ and although our titers are commensurate with this benchmark, morphine is synthesized several enzymatic steps downstream of (*S*)-reticuline. The efficiency of these reactions within a high titer production host remains unknown, yet the recent discovery of dedicated thebaine and neopinone biosynthetic enzymes^43,44^ primes our engineered yeast for production of opioid pharmaceuticals at scale. Beyond BIAs, our microbial production platform also enables the production of diverse THIQ molecules and could be leveraged to identify new medicines derived from the THIQ structural moiety.

Owing to their reactivity and toxicity within biological systems, microorganisms rapidly transform aldehydes to acids or alcohols^45^. Our initial BIA-producing strains were found to be limited by 4-HPAA, which is efficiently converted to tyrosol or 4-HPAC by a previously unidentified consortium of functionally redundant enzymes^15,46^. Many prior reports have ascribed the ADH family of predominantly NADH-dependent dehydrogenases to fusel alcohol formation, yet limited data has been set forth firmly linking these enzymes to the Ehrlich pathway^27,47^. We have resolved this long-standing issue by identifying a specific subset of NADPH-dependent enzymes implicated in tyrosol formation (Ari1, Adh6, Ypr1, Ydr541c, Aad3, and Gre2), underscoring tremendous redundancy in Ehrlich pathway oxidoreductases. To our knowledge, none of these 4-HPAA reductases have been previously assayed on 4-HPAA, while sparse evidence exists for their activities on other Ehrlich pathway aldehydes, such as Ypr1 on 2-MB^48^ and Gre2 on 3-MB^49^. In line with these reports, we showed that several 4-HPAA reductases are involved in the catabolism of other Ehrlich pathway amino acids, as their inactivation triggered the biosynthesis of THIQs derived from l-phenylalanine, l-tryptophan, and l-leucine. Abolishing tyrosol synthesis prompted us to also identify the major 4-HPAC-forming enzyme, a role we assigned to the Hfd1 aldehyde-dehydrogenase. This finding again contrasts prior reports in which the ALD family of enzymes are presumed to be the sole determinants of fusel acid formation in yeast^27^. Our work has implications within the food and beverage industries, in which production of fusel products is a key determinant of flavor profiles^27^. More broadly, the capacity of our engineered strain to synthesize high levels of both aliphatic and aromatic aldehydes suggests that it could be repurposed to produce diverse compounds derived from carbonyl intermediates, such as biofuels, fragrances, and chemotherapeutics^27,50–52^.

By diverting carbonyl intermediates of the yeast Ehrlich pathway to THIQ formation, our production host enables the synthesis of diverse privileged structures. We exploited this activity to build a suite of THIQ analogs by supplying both aromatic and aliphatic building blocks to our engineered host. We directly confirmed the identity of the substituted THIQ derived from supplemented l-DOPA by comparing its fragmentation spectrum to that of authentic norlaudanosoline standard. The identities of the remaining substituted THIQs synthesized in this study have yet to be unequivocally confirmed, yet we provide substantial independent data corroborating their presumed identities. All of the substituted THIQs synthesized herein exhibited a shift in retention time relative to the canonical BIA structures [(*S*)-norcoclaurine or (*S*)-*N*-methylcoclaurine] and yielded a mass accuracy of <5.0 parts per million based on the presumed exact masses. Fragmentation of these structures yielded key fragments indicative of the THIQ moiety, implying that these compounds arise from dopamine. Further, the retention time of peaks corresponding to alkyl-substituted THIQs **31**, **35**, **39**, and **43** increased incrementally, which reflects the relative hydrophobicity of the presumed structures. Lastly, inactivation of l-phenylalanine and l-tryptophan biosynthesis pathways dramatically decreased peaks corresponding to THIQ products **16** and **19**, respectively, providing further evidence that these structures arise from aromatic amino acids.

To our knowledge many of the presumed THIQ structures synthesized in this study have not been observed in nature and thus our work broadens the chemical space occupied by natural THIQ products. It is noteworthy that the isobutyl-substituted THIQ (**22**) derived from l-leucine has been identified in the senita cactus (*Lophocereus schotti*) where it forms the basis of the methylated alkaloid lophocerine^40^. Our (*S*)-reticuline production host recapitulated this natural pathway, as implementation of *Ps*6OMT and *Ps*CNMT resulted in de novo synthesis of lophocerine. We also observed the formation of salsolinol and its methylated derivatives, demonstrating that diverse THIQ scaffolds can be furnished with functional groups using canonical BIA tailoring enzymes. Furthermore, the THIQ scaffold formed from PAA (**16**) possesses the core BIA skeleton, indicating that the benzyl moiety of BIAs can also derive from l-phenylalanine. Products arising from **16** would lack hydroxylation at the 4ʹ position of the benzyl substituent that originates from 4-HPAA and is present in nearly all BIA structures characterized to date. In this context, sacred lotus (*Nelumbo nucifera*) synthesizes aporphine alkaloids possessing an unsubstituted benzyl moiety^53^ and thus our work exposes a plausible biosynthetic route to these distinct BIAs.

In summary, we engineered a yeast host capable of synthesizing gram per liter titers of the central BIA intermediate (*S*)-reticuline. This metabolite is a direct precursor to all natural BIAs, including the morphinan family, providing a platform for the microbial synthesis of natural opiates and their semi-synthetic derivatives. Microbial biosynthesis also provides the opportunity to access thousands of untapped metabolites that are present at low levels in source plants, a goal that is now within reach given our improvements in yeast THIQ biosynthesis. Finally, linking plant BIA metabolism to our repurposed Ehrlich pathway afforded yeast with the capacity to synthesize diverse THIQ scaffolds. The THIQ motif is a privileged substructure that primes many natural products for bioactivity and thus harnessing this activity expands the diversity of THIQ alkaloids beyond the canonical l-tyrosine pathway and will bring about the development of alkaloids with new pharmacological properties.

## Methods

### Strains and growth media

The quadruple auxotrophic *S. cerevisiae* strain BY4741 (*MATa his3Δ1 leu2Δ0 met15Δ0 ura3Δ0*) was employed in this study. The dopamine-producing strain derived from BY4741 (strain 1373) was utilized as the basis for a yeast BIA platform strain. Yeast cultures were grown in YPD medium (10 g L^−1^ Bacto Yeast Extract, 20 g L^−1^ Bacto peptone, 20 g L^−1^ glucose). Transformed cells were selected on YPD agar containing 200 μg mL^−1^ hygromycin B, 400 μg mL^−1^ G418, or a combination of both antibiotics (200 μg mL^−1^ each). Selection using auxotrophic markers was performed in synthetic complete (SC) medium [6.7 g L^−1^ Difco Yeast Nitrogen Base (YNB) without amino acids, 1.62–1.92 g L^−1^ Drop-out Medium Supplements (Millipore-Sigma) minus appropriate amino acids, 20 g L^−1^ glucose). Strains in which prototrophy was restored were selected on YNB medium (6.7 g L^−1^ Difco YNB, 20 g L^−1^ glucose). Oligonucleotides, plasmids, and strains employed in this work are provided in Supplementary Data 2, Supplementary Table 2, and Supplementary Data 3, respectively.

### Yeast strain construction

All genetic modifications to yeast were made via CRISPR-Cas9-mediated genomic integration^54,55^ and in vivo DNA assembly^56^. Cas9 and genomic RNA (gRNA) were delivered to yeast using pCas-G418^55^ or a hygromycin-resistance derivative (pCas-Hyg) constructed herein. Linear gRNA cassettes were retargeted by PCR and assembled using in vivo gap repair with a linear PCR-generated pCas backbone^54^. Approximately 100 ng of linear pCas was combined with 250 ng of linear gRNA cassette and 500–1000 ng of total repair DNA in a standard 50 μL lithium acetate transformation. Cells were heat-shocked at 42 °C for 30 min, recovered for 16 h, and plated onto YPD plates containing appropriate antibiotics. Both linear pCas-G418 and pCas-Hyg plasmids were used for transformations involving complex multi-part DNA assemblies. Design of gRNAs was performed by selecting top hits common to both CCTop^57^ and a yeast CRISPRi webtool^58^. Chromosomal loci for DNA integration were selected from previous studies^29,59–61^. Gene expression cassettes and genomic integration sites utilized in this work are listed in Supplementary Tables 3 and 4, respectively. Synthetic DNAs employed in this study are provided in Supplementary Data 4.

Yeast genes targeted for deletion were replaced with a synthetic DNA landing pad (LP5.T3) possessing a unique Cas9 target site (T3) for subsequent genomic integration of additional copies of the *NdNCSΔN*_*20*_ gene. Seven of the eight copies of *NdNCSΔN*_*20*_ were later deleted in one genome editing event using the *NdNCS* (T_*PGI1*_-LP5) gRNA and a single chromosomal LP5.T3 donor in the *aad3*Δ locus. The remaining *NdNCSΔN*_*20*_ copy was deleted from site FgF20 using an exogenous LP5.T3 donor. *ALD4* was initially deleted using LP5.T3 and reintroduced in later strains along with its cognate promoter and terminator at a different locus (308a).

### Growth curves

Growth curves were generated in triplicate in 96-well microtiter plates containing 180 μL of 1 × SC medium containing 2% sucrose. Cultures were inoculated using saturated overnight cultures to an initial OD_595_ of roughly 0.2 (50-fold dilution) and wrapped in Parafilm to minimize evaporation. Absorbance readings were taken at 595 nm every 20 min with a Sunrise absorbance microplate reader (Tecan) over the course of 2–3 days. Maximum specific growth rates (*μ*_max_, h^−1^) were determined from triplicate cultures and were based on OD_595_ readings.

### Fluorescence microscopy

To visualize GFP-tagged proteins, cells from overnight cultures were back-diluted 50× into fresh SC medium and incubated at 30 °C and 200 r.p.m. for 4–6 h. Cells were washed with water and mounted unfixed on microscope slides. Images were captured using a Nikon Ti microscope with a 100× PlanAPO lens (NA 1.49). Cells were illuminated using high inclination laminated optical sheet TIRF illumination with 488 nm lasers, and its respective filter cube (Chroma). Images are of single planes. Image processing was done using Fiji (NIH).

### Microtiter plate assay for BIA and fusel metabolites

Colonies were picked in triplicate into 0.5 mL of 2× SCS medium (13.4 g L^−1^ Difco Yeast Nitrogen Base (YNB) without amino acids, 2× Drop-out Medium Supplements (Millipore-Sigma) minus appropriate amino acids, 40 g L^−1^ sucrose) within 96-well deep well plates. Following 16–24 h of growth, saturated cultures were back-diluted 50× into 0.5 mL of fresh 2× SCS medium in 96-well deep well plates. Cultures were grown at 30 °C with shaking at 350 r.p.m. Following 72–96 h of growth, OD_600_ measurements were taken and culture broth was stored at −20 °C for subsequent analysis by LC–MS.

### Fed-batch cultivation of strain LP507 in a mineral medium

Controlled fed-batch fermentations were carried out in 3 L BioBundle fermentors (Applikon). Cultivation temperature was maintained at 30 °C and pH was kept at 4.5 by titration with 25% NH_3_ in H_2_O. Dissolved oxygen was maintained at 30% of air saturation by automatically adjusting the stirring rate (aeration rate 1.0 L min^−1^). Off-gas composition (concentration of O_2_ and CO_2_) was analyzed using a Tandem Multiplex gas analyzer (Magellan BioTech). Bioreactor inoculum was generated in two 250 mL shake flasks containing 50 mL of SC-His medium, grown for 24 h at 30 °C. Cells were washed and suspended in 0.9% NaCl, and used to inoculate (OD_600_ = ~0.1) 1 L of batch medium (40 g sucrose, 2.5 g KH_2_PO_4_, 6.0 g (NH_4_)_2_SO_4_, 1.0 g MgSO_4_·7H_2_O, 5 mL vitamin stock, and 5 mL trace element stock per liter). Vitamin and trace element stock solutions were based on a previous report^62^. The culture was operated in batch mode until sucrose was exhausted (36 h), followed by fed-batch phase with automated 10 g L^−1^ sucrose pulses (Supplementary Fig. 8). Feeding medium contained 500 g sucrose, 20.8 g KH_2_PO_4_, 5 g (NH_4_)_2_SO_4_, 8.3 g MgSO_4_·7H_2_O, 5 g K_2_SO_4_, 20 mL vitamin stock, and 20 mL trace element stock per liter. Samples were collected every 12 h for a total of 6 days. Cell dry weight (in g L^−1^) was calculated using a conversion factor of 0.59 g L^−1^ per OD_600_ (determined gravimetrically).

### THIQ synthesis assay from supplemented amino acids

For growth on individual amino acids as the major source of nitrogen, reticuline-producing strain LP501 was transformed with pHUM^63^ to complement His and Met auxotrophies. Colonies were first picked in triplicate into 0.5 mL of 2× YNB (without amino acids and ammonium sulfate) containing 40 g L^−1^ sucrose and 1 g L^−1^ urea. Following 24 h of growth, saturated cultures were back-diluted 40× into 0.5 mL of fresh 2× YNB (without amino acids and ammonium sulfate) containing 40 g L^−1^ sucrose within 96-well deep well plates. Cultures of strain LP501 harboring pHUM were also supplemented with 0.076 g L^−1^ each of l-phenylalanine and l-tryptophan due to *pha2*Δ and *trp3*Δ mutations. For THIQ synthesis, cultures were supplemented with individual amino acids to a final concentration of 0.45 g L^−1^ (l-tyrosine), 2 g L^−1^ (l-2-aminoheptanoic acid, l-tryptophan, and l-DOPA), 3 g L^−1^ (l-norleucine), or 5 g L^−1^ (l-2-aminobutyrate, l-leucine, l-methionine, l-norvaline, and l-phenylalanine). Ascorbic acid (10 mM) was added to cultures supplemented with l-tryptophan, l-tyrosine, or l-DOPA. Cultures were grown at 30 °C with shaking at 350 r.p.m. Following 120 h of growth, OD_600_ measurements were taken and culture broth was stored at –20 °C for subsequent analysis by LC–MS.

### LC–MS and high-performance liquid chromatography-ultraviolet (HPLC-UV) analysis of metabolites

Dopamine, BIA, and other THIQ products from microtiter plate cultures were analyzed using HPLC-FT-MS. Metabolites were extracted from culture broth containing cells and growth medium. For early strains, 25 μL of culture broth was combined with 100 μL of cold 100% acetonitrile (ACN) and 542 μL of 0.123% formic acid was added to give a final concentration of 15% ACN and 0.1% formic acid. For more productive BIA strains and fed-batch cultures, an additional tenfold dilution of samples was performed. Samples were centrifuged at 4000 RCF and 10 μL of extracted culture supernatant was separated on a 1290 Infinity II LC system (Agilent Technologies) with a Zorbax Rapid Resolution HT C18 column (30 × 2.1 mm, 1.8 μm; Agilent Technologies). Metabolites were separated using the following gradient: 2% B to 10% B from 0 to 4 min (0.3 mL min^−1^), 10% B to 85% B from 4 to 6 min (0.3 mL min^−1^), held at 85% B from 6 to 7 min (0.3 mL min^−1^), 85% B to 2% B from 7 to 7.1 min (0.3 mL min^−1^), and held at 2% B from 7.1 to 9 min (0.45 mL min^−1^). Solvent A was 0.1% formic acid in water and solvent B was 0.1% formic acid in 100% ACN. Following separation, eluent was injected into an LTQ-FT-MS (Thermo Fisher Scientific) using 100 to 400 *m/z* scanning range in positive mode. Resolution, capillary voltage, and source temperature were set to 100,000, 5 kV, and 350 °C, respectively. FT-MS data was processed and manipulated using Xcalibur Qualitative Analysis software (Thermo Fisher Scientific).

Samples producing substituted THIQs from supplemented amino acids were analyzed using an Agilent 6545 quadrupole time-of-flight MS (QTOF-MS; Agilent Technologies) equipped with a Zorbax Eclipse Plus C18 column (50 × 2.1 mm, 1.8 μm; Agilent Technologies) and using the aforementioned gradient conditions. The sample tray and column compartment were set to 4 °C and 30 °C, respectively. The sheath gas flow rate and temperature were adjusted to 10 L min^−1^ and 350 °C, respectively, while drying and nebulizing gases were set to 12 L min^−1^ and 55 psig, respectively. The drying gas temperature was set to 325 °C. QTOF data was processed and manipulated using Agilent MassHunter Qualitative Analysis software.

Dopamine and BIAs from fermentor samples and fusel products from both microtiter plate and fermentor cultures were analyzed and quantified using HPLC-UV. Equal volumes of culture broth and 100% ACN containing 0.1% trifluoroacetic acid (TFA) were combined and samples were centrifuged at 4000 RCF. Additional dilutions were performed as necessary. Five microliters of extracted broth was separated on an Agilent 1200 HPLC system equipped with an Eclipse XDB-C18 column (150 × 4.6 mm, 5 μm, Agilent Technologies). Metabolites were separated using a flow rate of 1 mL min^−1^ and the following gradient: 5% B to 20% B from 0 to 10 min, 20% B to 50% B from 10 to 15 min, 50% B to 95% B from 15 to 15.1 min, and held at 95% B from 15.1 to 25 min. Solvent A was 0.1% TFA in water and solvent B was 0.1% TFA in 100% methanol. Tyrosol and 4-HPAC were detected at 276 nm; dopamine, (*S*)-norcoclaurine, and (*S*)-reticuline at 280 nm.

Chiral analysis of norcoclaurine was performed by modifying an existing method^15^. Culture supernatant from strain LP478 was centrifuged to remove debris and diluted 1:50 in H_2_O. Ten microliters was loaded onto a SHODEX ORPak CDBS-453 column and separated chromatographically using a 1290 Infinity II HPLC (Agilent Technologies) and the following gradient: 0–13 min, 5% B; 14–17 min, 95% B; 18–30 min, 5% B where Buffer A was 0.1% formic acid and Buffer B was 0.1% formic acid in ACN. Flow rate was 0.25 mL min^−1^ and temperature was held constant at 25 °C. Compounds eluting from the column were analyzed with a 6560 Ion Mobility QTOF mass spectrometer (Agilent Technologies) with the following settings: gas temp, 325 °C; drying gas, 12 L min^−1^; nebulizer 55 psig; sheath gas temp 350 °C; sheath gas flow 10 L min^−1^; capillary voltage 4000 V; nozzle voltage 500 V; fragmentor 400 V. Norcoclaurine was identified by exact mass (*m*/*z* 272.128 [M + H]^+^). Retention time of (*S*)- and (*R*)-norcoclaurine was established by analysis of an (*S*)-norcoclaurine authentic standard (Toronto Research Chemicals Inc.) and racemic norcoclaurine, which was spontaneously condensed from dopamine (Sigma) and 4-HPAA (gift from P. Facchini, University of Calgary) in a KH_2_PO_4_/ACN buffer^25^.

### Statistical analyses

All numerical values are depicted as means ± s.d. Statistical differences between control and derivative strains were assessed via two-tailed Student’s *t*-tests assuming equal variances using Excel (Microsoft) or Prism (GraphPad Software Inc.). In all cases, *P*-values < 0.05 were considered significant.

### Reporting summary

Further information on research design is available in the Nature Research Reporting Summary linked to this article.

## Supplementary information

Supplementary Information

Reporting Summary

Description of Additional Supplementary Files

Supplementary Data 1

Supplementary Data 2

Supplementary Data 3

Supplementary Data 4

## Data Availability

Data supporting the findings of this work are available within the paper and its Supplementary Information files. A reporting summary for this Article is available as a Supplementary Information file. The datasets generated and analyzed during the current study are available from the corresponding author upon request. The source data of Figs. 1b, 2b, c and 3c, as well as Supplementary Figs. 1b, 2–7, 8a, 9–12, 14, and 15 are provided as a Source Data file.

## Source data

Source Data
