# Supplementary material for: A yeast platform for high-level synthesis of tetrahydroisoquinoline alkaloids
Source: Nat Commun. 2020 Jul 3;11:3337. doi: 10.1038/s41467-020-17172-x (PMC7335070; doi:10.1038/s41467-020-17172-x)
Supplement: Supplementary file 1 — Supplementary Information [file 41467_2020_17172_MOESM1_ESM.pdf]

A yeast platform for high-level synthesis of tetrahydroisoquinoline alkaloids

Pyne *et al*.

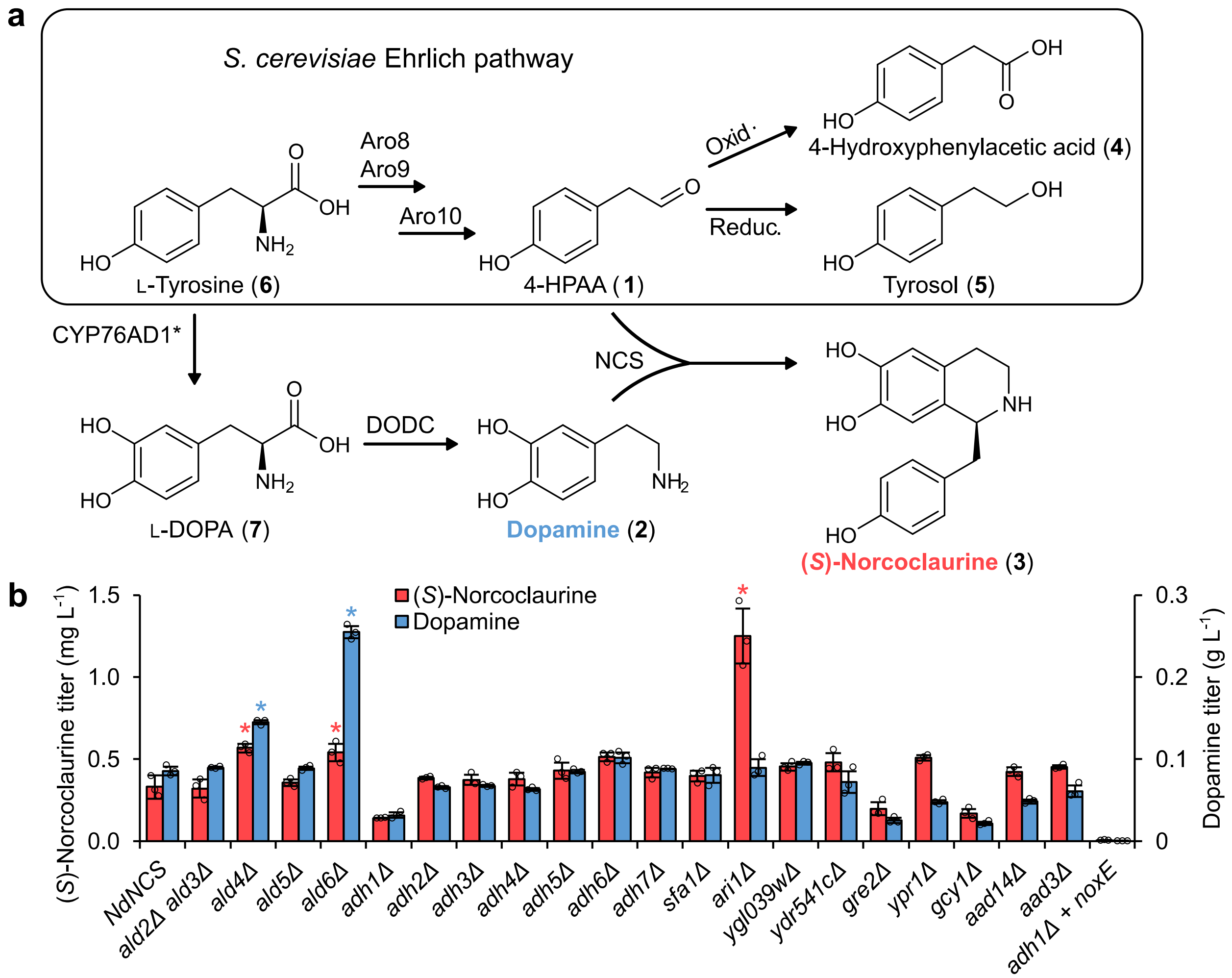

Supplementary Figure 1. Enhancing 4-HPAA substrate supply through inactivation of host oxidoreductases. (**a**) 4-HPAA is produced natively from L-tyrosine via the Ehrlich pathway where it is converted to 4-hydroxyphenylacetic acid (4-HPAC) or tyrosol. Implementing NCS and a heterologous dopamine biosynthesis pathway diverts 4-HPAA to (*S*)-norcoclaurine formation. (**b**) Dopamine and (*S*)-norcoclaurine titers in culture supernatants of single gene deletion strains as measured by LC-MS. Deletions were performed in a strain harboring a single gene copy of *NdNCS*. *ALD2* and *ALD3* were deleted in conjunction due to proximity in the yeast genome. An NADH oxidase gene (*noxE*) was expressed in the *adh1*Δ mutant to improve growth[1]. Asterisk (*) denotes a significant increase ($P < 0.05$) in titer relative to the parent strain. Statistical differences between control and derivative strains were tested using two-tailed Student's *t*-test. Error bars represent the mean ± s.d. of n=3 independent biological samples. Source data underlying Supplementary Figure 1b are provided in a Source Data file.

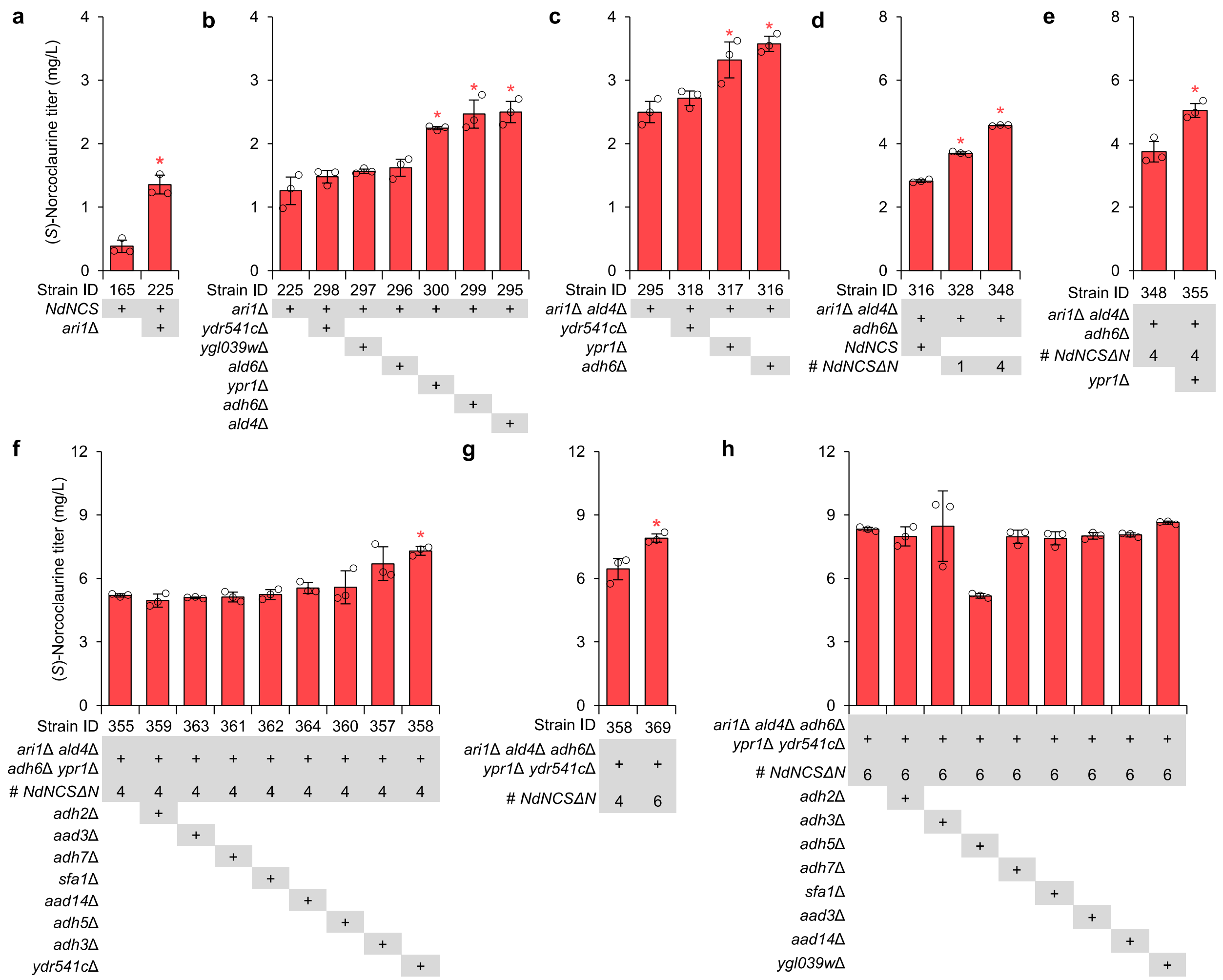

Supplementary Figure 2. Combinatorial gene deletion analysis to improve (*S*)-norcoclaurine production. (**a** to **h**) Successive rounds of strain engineering were performed to identify the optimal combination of oxidoreductase gene deletions. NCS activity was also improved through truncation of *NdNCS* (**d**) and increasing copy number of $NdNCS\Delta N_{20}$ (**d** and **g**). Error bars represent the mean ± s.d. of n=3 independent biological samples. Asterisks (*) denote a significant increase ($P < 0.05$) in (*S*)-norcoclaurine production relative to the parent strain. Statistical differences between control and derivative strains were tested using two-tailed Student's *t*-test. Source data are provided in a Source Data file.

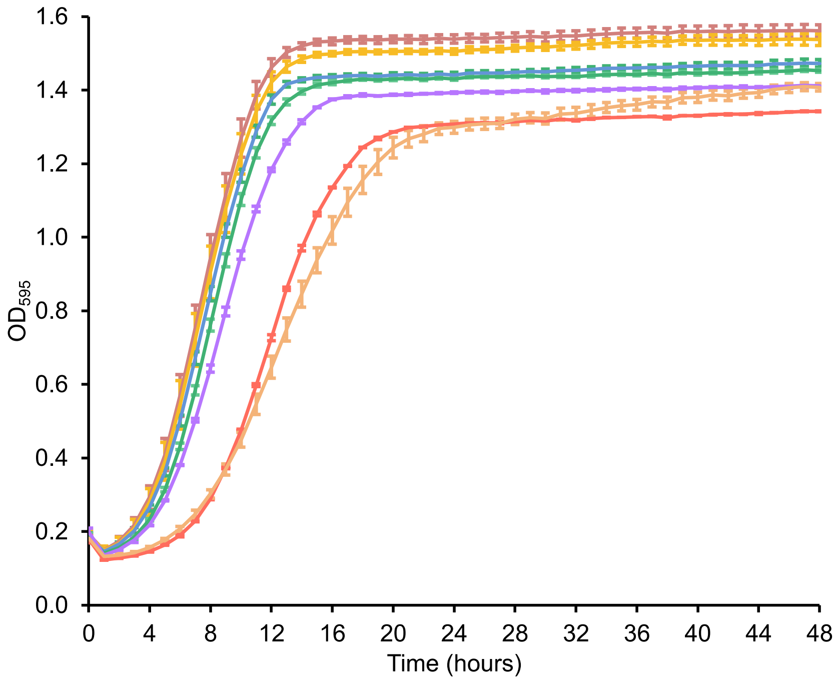

Strain ID	BY4741	1373	LP165	LP358	LP376	LP379	LP478
Dopamine pathway	-	+	+	+	+	+	+
NdNCS	-	-	+	+	+	+	+
*ari1*Δ *adh6*Δ	-	-	-	+	+	+	+
*ald4*Δ	-	-	-	+	+	+	-
# *NdNCS*ΔN_{20}	0	0	0	6	6	6	0
*ypr1*Δ *ydr541c*Δ	-	-	-	+	+	+	+
P_{TDH3}-*TYR1*, *ARO7*^{FBR}, *ARO10*	-	-	-	-	+	+	+
*pha2*Δ *trp3*Δ	-	-	-	-	-	+	+
P_{TDH3}-*ARO2*	-	-	-	-	-	-	+
*aad3*Δ	-	-	-	-	-	-	+
# *CjNCS*ΔN_{35}	0	0	0	0	0	0	2
P_{TEF1}-*CYP76AD5*	-	-	-	-	-	-	+
μ_{max} (h^{-1})	0.32	0.32	0.30*	0.30	0.27*	0.24*	0.20*
s.d. (h^{-1})	0.0036	0.0040	0.0025	0.0031	0.0029	0.0017	0.0030

Supplementary Figure 3. Growth curves of key (*S*)-norcoclaurine-producing strains. Growth curves, maximum specific growth rates (μ_{max}, h^{-1}), and relevant genotypes of intermediate (*S*)-norcoclaurine-producing strains are shown. Strain 1373 derives from BY4741 and possesses the dopamine pathway (*CYP76AD1*, *DODC*, and *ARO4*^{FBR}), yet lacks an NCS biosynthetic enzyme. Introduction of *NdNCS* (strain LP165) leads to a 6% decline in maximum specific growth rate, while growth was unaffected by deletion of five oxidoreductases (*ari1*Δ *adh6*Δ *ald4*Δ *ypr1*Δ *ydr541c*Δ; strain LP358). Upregulation of L-tyrosine and Ehrlich pathways (strain LP376) and introduction of L-phenylalanine and L-tryptophan auxotrophies (strain LP379) led to further declines in maximum specific growth rate (10% and 11%, respectively). Overall strain LP478 exhibited a 38% decrease in maximum specific growth rate relative to the BY4741 parent. Overnight cultures were back-diluted to an initial OD_{595} of roughly 0.2 (50-fold dilution) and grown in 180 µL of 1× SC medium containing 2% sucrose. Error bars represent the mean ± s.d. of n=3 independent biological samples. Asterisks (*) denote a significant decrease ($P < 0.05$) in maximum specific growth rate relative to the corresponding precursor strain. Statistical differences between control and derivative strains were tested using two-tailed Student's *t*-test. Source data are provided in a Source Data file.

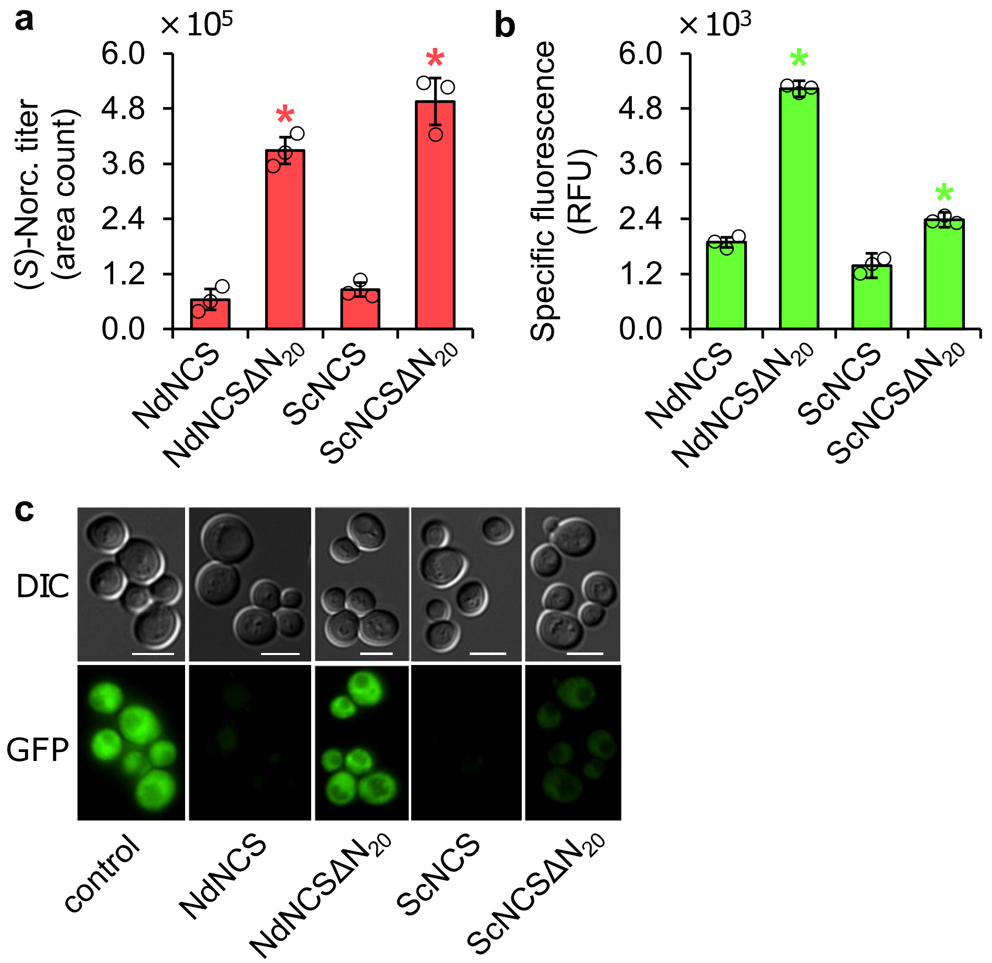

Supplementary Figure 4. N-Terminal truncation of *Nd*NCS or *Sc*NCS improves production of (*S*)-norcoclaurine. *Nd*NCS and *Sc*NCS were truncated by removing 20 N-terminal amino acids, yielding *Nd*NCSΔN_{20} and *Sc*NCSΔN_{20}, respectively. (**a**) (*S*)-Norcoclaurine titer increases following truncation of *Nd*NCS and *Sc*NCS. (**b**) Specific GFP fluorescence (normalized to culture OD_{600}) increases following truncation of *Nd*NCS and *Sc*NCS. The C-termini of *Nd*NCS, *Nd*NCSΔN_{20}, *Sc*NCS, and *Sc*NCSΔN_{20} were fused with GFP. Overnight cultures were back-diluted 50× and grown in 0.5 mL of 2× SC medium for approximately 6 hours. Error bars represent the mean ± s.d. of n=3 independent biological samples. (**c**) N-terminal truncation of *Nd*NCS and *Sc*NCS improves gene expression or enzyme solubility in yeast. Cells of GFP-tagged *Nd*NCS, *Nd*NCSΔN_{20}, *Sc*NCS, and *Sc*NCSΔN_{20} were visualized using confocal fluorescence microscopy. Control cells harbor GFP without NCS. Scale bars represent 5 μm. Microscopy samples were prepared in duplicate and yielded similar results. Asterisks (*) denote a significant increase ($P < 0.05$) in (*S*)-norcoclaurine production or GFP fluorescence relative to the parent strain. Statistical differences between control and derivative strains were tested using two-tailed Student's *t*-test. Source data are provided in a Source Data file.

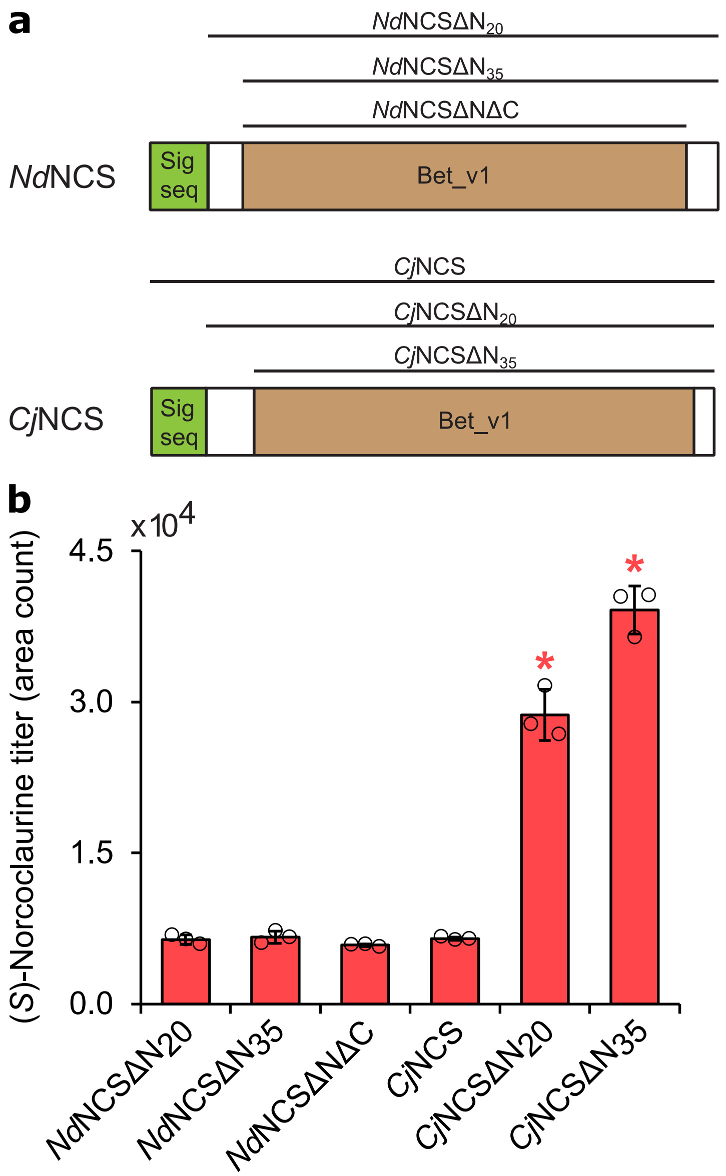

Supplementary Figure 5. Truncation of *Cj*NCS improves production of (*S*)-norcoclaurine. *Nd*NCS and *Cj*NCS were truncated by removing various-sized N-terminal and C-terminal regions. (**a**) Structure of N- and C-terminal truncations of *Nd*NCS and *Cj*NCS proteins. Bet_v1 is the putative core NCS catalytic domain required for activity and Sig seq refers to predicted N-terminal signal sequences for targeting *Nd*NCS and *Cj*NCS to subcellular organelles in their respective plant species. (**b**) (*S*)-Norcoclaurine titer increases following truncation of *Cj*NCS to the core Bet_v1 domain. Strains were grown in 0.5 mL of 2× SC medium for 72. Error bars represent the mean ± s.d. of n=3 independent biological samples. Asterisks (*) denote a significant increase ($P < 0.05$) in (*S*)-norcoclaurine production relative to the strain harboring *Nd*NCSΔN_{20}. Statistical differences between control and derivative strains were tested using two-tailed Student's *t*-test. Source data are provided in a Source Data file.

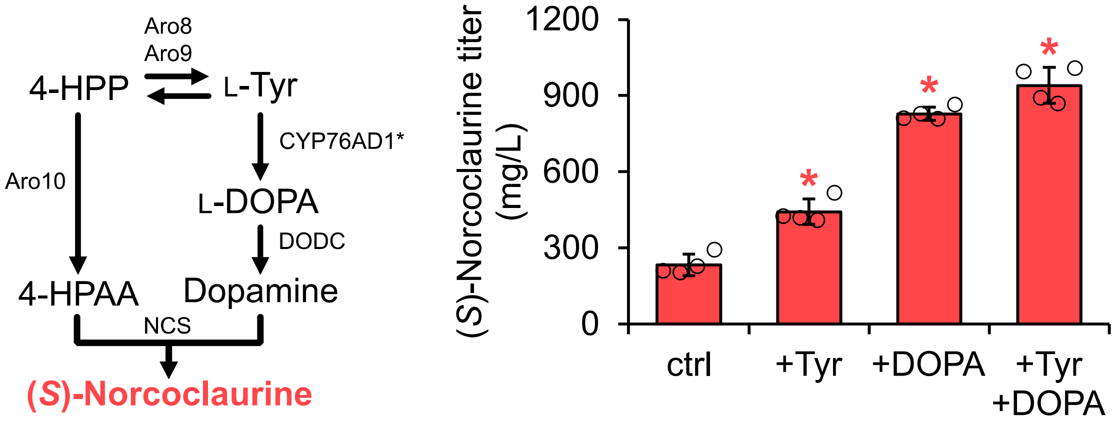

Supplementary Figure 6. Supplementation of L-DOPA to strain LP412 improves (*S*)-norcoclaurine production. Exogenously supplied L-DOPA is converted directly to dopamine, whereas L-tyrosine is converted to both 4-HPAA and dopamine. Cultures of strain LP412 were supplemented with 2.5 mM L-tyrosine, 5 mM L-DOPA, or a combination of both amino acids and grown in 0.5 mL of 2× SC medium for 72 hours. Ten mM sodium ascorbate was added to all cultures to limit oxidation of aromatic amino acids. Error bars represent the mean ± s.d. of n=4 independent biological samples. Asterisks (*) denote a significant increase ($P < 0.05$) in (*S*)-norcoclaurine production relative to the control culture. Statistical differences between control and derivative strains were tested using two-tailed Student's *t*-test. Source data are provided in a Source Data file.

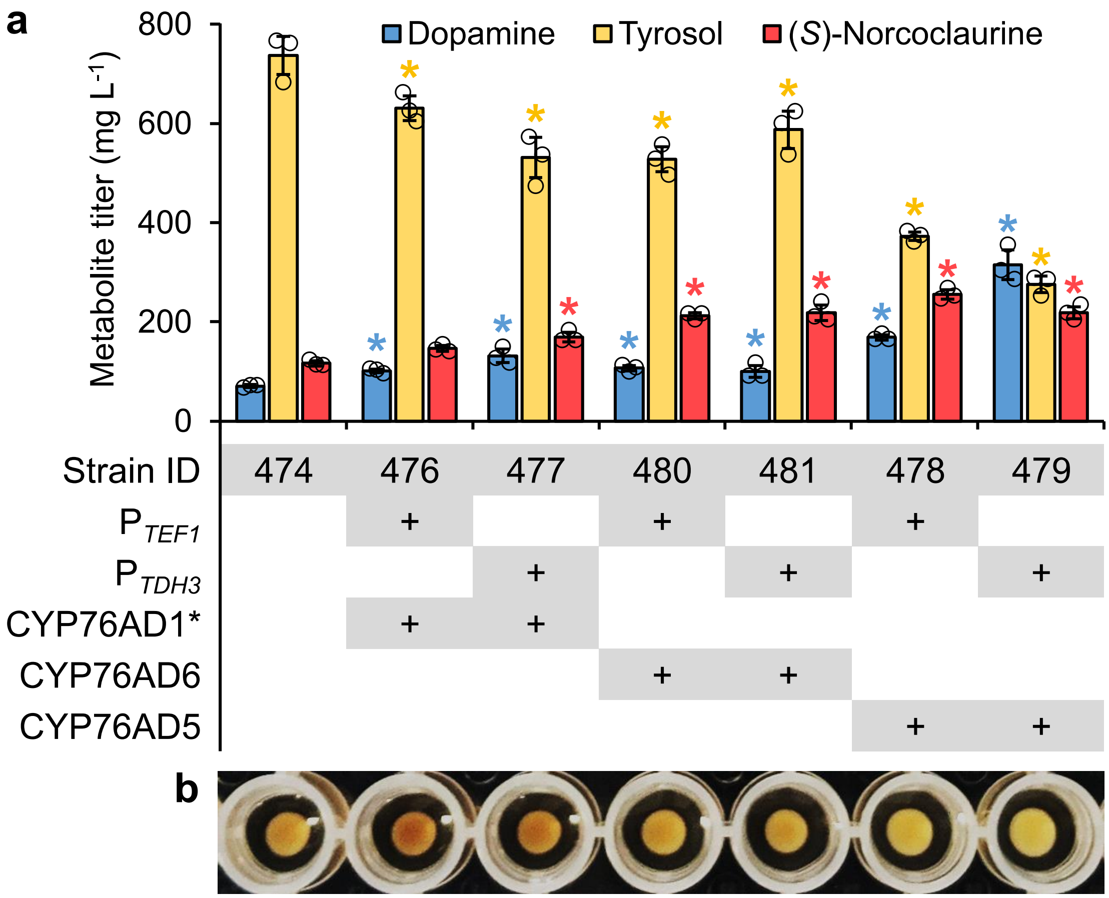

Supplementary Figure 7. Expression of *CYP76AD5* or *CYP76AD6* enhances (*S*)-norcoclaurine biosynthesis. Tyrosine hydroxylase variants (*CYP76AD1**, *CYP76AD6*, or *CYP76AD5*) were integrated into strain LP474 and expressed from one of two promoters (P_{TEF1} or P_{TDH3}). Strain LP474 and its derivatives contain an existing copy of P_{TDH3}-*CYP76AD1** ($CYP76AD1^{W13L\ F309L}$). (**a**) Implementation of *CYP76AD5*, *CYP76AD6*, or an additional copy of *CYP76AD1** yields a range of tyrosol, dopamine, and (*S*)-norcoclaurine titers. CYP76AD5 is a more active enzyme than CYP76AD1* and CYP76AD6 (ref. [2]). P_{TDH3} is a stronger promoter than P_{TEF1} (ref. [3,4]). Expression of *CYP76AD5* from P_{TEF1} yielded the highest (*S*)-norcoclaurine titer of all strains assayed, while its expression from P_{TDH3} yielded the highest dopamine titer and the lowest concentration of tyrosol. Error bars represent the mean ± s.d. of n=3 independent biological samples. Asterisks (*) denote a significant increase or decrease ($P < 0.05$) in metabolite production relative to strain LP474. Statistical differences between control and derivative strains were tested using two-tailed Student's *t*-test. (**b**) Pigmentation of cells expressing *CYP76AD1**, *CYP76AD6*, or *CYP76AD5*. CYP76AD1 and its engineered variant (CYP76AD1*) possess DOPA oxidase side activity not observed in CYP76AD5 and CYP76AD6 (ref. [2]), which results in the accumulation of melanin, a brown pigment[5]. Strains for pigmentation and metabolite production assays were grown in 0.5 mL of 2× SC medium for 96 hours. Source data are provided in a Source Data file.

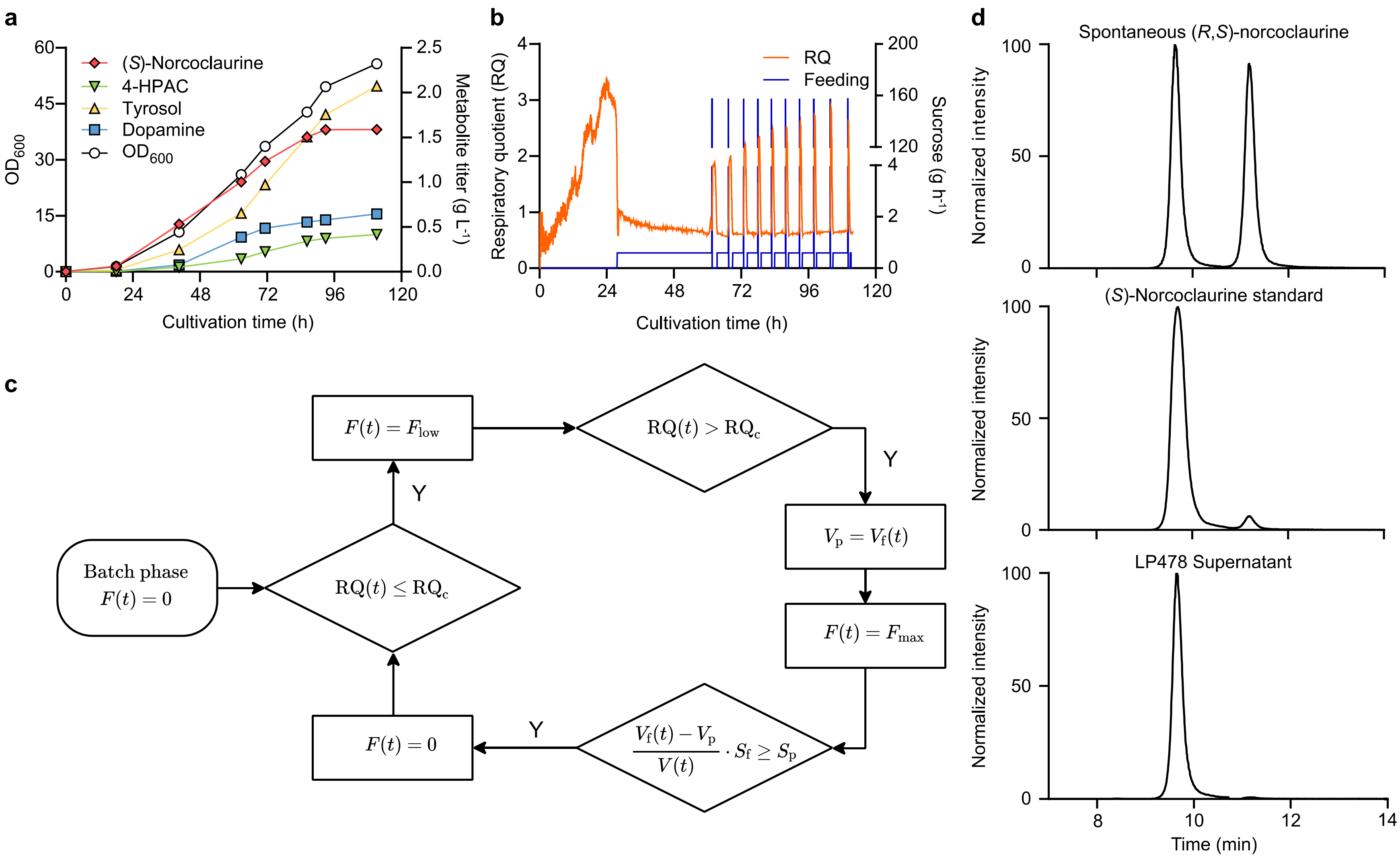
a
(S)-Norcoclaurine
4-HPAC
Tyrosol
Dopamine
OD600
Metabolite titer (g L-1)
Cultivation time (h)
b
Respiratory quotient (RQ)
RQ
Feeding
Sucrose (g h-1)
Cultivation time (h)
c
Batch phase
$F(t) = 0$
$RQ(t) \le RQ_c$
Y
$F(t) = F_{low}$
$RQ(t) > RQ_c$
Y
$V_p = V_f(t)$
$F(t) = F_{max}$
$\frac{V_f(t) - V_p}{V(t)} \cdot S_f \ge S_p$
Y
$F(t) = 0$
d
Spontaneous (R,S)-norcoclaurine
(S)-Norcoclaurine standard
LP478 Supernatant
Normalized intensity
Time (min)

Supplementary Figure 8. Cultivation of strain LP478 in a pulsed fed-batch fermentor. (**a**) Growth of biomass (OD_{600}) and accumulation of metabolites. Fed-batch cultivation was performed as described in the Methods section with the following changes. Batch medium was supplemented with 1.92 g L^{-1} Drop-out Medium Supplements without histidine, 0.076 g L^{-1} L-tryptophan, and 0.152 g L^{-1} L-phenylalanine. Feeding medium contained 360 g sucrose, 15 g KH_2PO_4, 60 g $(NH_4)_2SO_4$, 6 g $MgSO_4 \cdot 7H_2O$, 4.16 g L-phenylalanine, 1.55 g L-tryptophan, 15 mL vitamin stock, and 15 mL trace element stock per liter. Culture pH was maintained at pH 4.5 by titration with 4 M NaOH. (**b**) Cells were grown in batch phase until exhaustion of sucrose (40 g L^{-1}), indicated by a rapid drop of respiratory quotient (RQ) value, triggering constant feeding of fed-batch medium (corresponding to 0.60 g h^{-1} sucrose). Constant feeding continued until the exhaustion of ethanol (indicated by an increase in RQ value) produced in the batch phase. Subsequently, a pulse of fed-batch medium (corresponding to 10 g L^{-1} sucrose) was rapidly fed into the reactor, after which the pump was stopped. Feeding (0.60 g h^{-1} sucrose) resumed after exhaustion of sucrose, and until consumption of ethanol, after which another pulse (10 g L^{-1} sucrose) was fed, continuing the cycle. (**c**) Logic chart of pulse feeding algorithm. $F(t)$, substrate (sucrose) feeding rate at time t; $RQ(t)$, on-line value of RQ at time t; RQ_c, trigger value of RQ; F_{low}, substrate feeding rate setpoint for constant feeding; F_{max}, substrate feeding rate setpoint for pulse feeding; $V_f(t)$, volume of feeding medium fed into the reactor at time t; V_p, feeding volume storage value; $V(t)$, current culture volume; S_f, concentration of sucrose in the feeding medium; S_p, target concentration of sucrose in the bioreactor after a pulse; t, time. (**d**) Chiral analysis of norcoclaurine produced by LP478. LC-MS chromatograms of (*R*,*S*)-norcoclaurine from spontaneously-condensed dopamine and 4-HPAA (top panel), an authentic (*S*)-norcoclaurine standard (middle panel), and supernatant from a fed-batch fermentor sample derived from strain LP478 (bottom panel). (*R*)- and (*S*)-enantiomers were separated using a chiral column, demonstrating that LP478 synthesizes exclusively (*S*)-norcoclaurine. Source data underlying Supplementary Figure 8a are provided in a Source Data file.

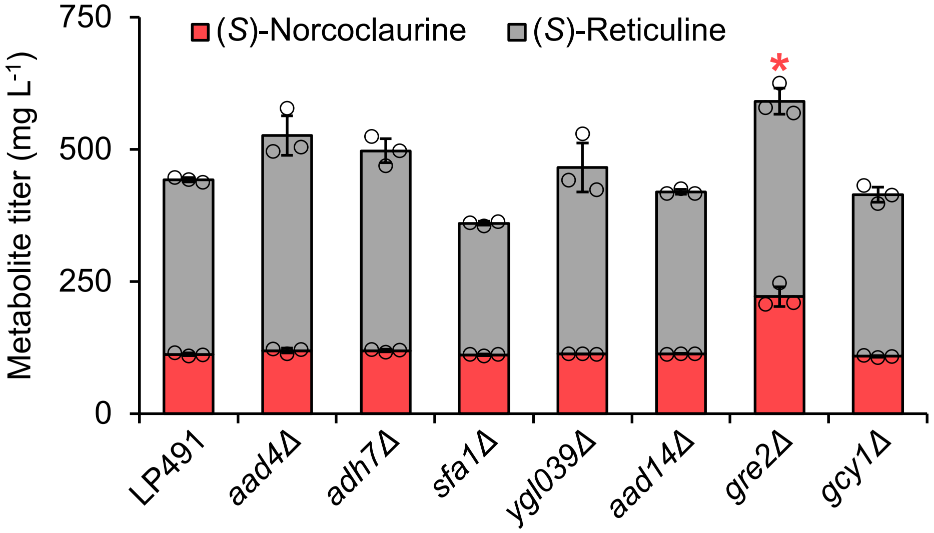

Supplementary Figure 9. Deletion of *GRE2* in strain LP491 increases BIA production in microtiter plate cultures. (*S*)-Norcoclaurine and (*S*)-reticuline titers in culture supernatants of strain LP491 containing deletions in oxidoreductase genes. Strain LP491 is an (*S*)-reticuline-producing strain containing deletions in five oxidoreductase genes (*ari1*Δ *adh6*Δ *ypr1*Δ *ydr541c*Δ *aad3*Δ). Deletion of *GRE2* facilitates a significant increase in (*S*)-norcoclaurine rather than (*S*)-reticuline production due to a presumed bottleneck in an (*S*)-reticuline pathway enzyme in microtiter plate cultures. Asterisk (*) denotes a significant increase ($P < 0.05$) in (*S*)-norcoclaurine titer relative to strain LP491. Statistical differences between control and derivative strains were tested using two-tailed Student's *t*-test. Error bars represent the mean ± s.d. of n=3 independent biological samples. Source data are provided in a Source Data file.

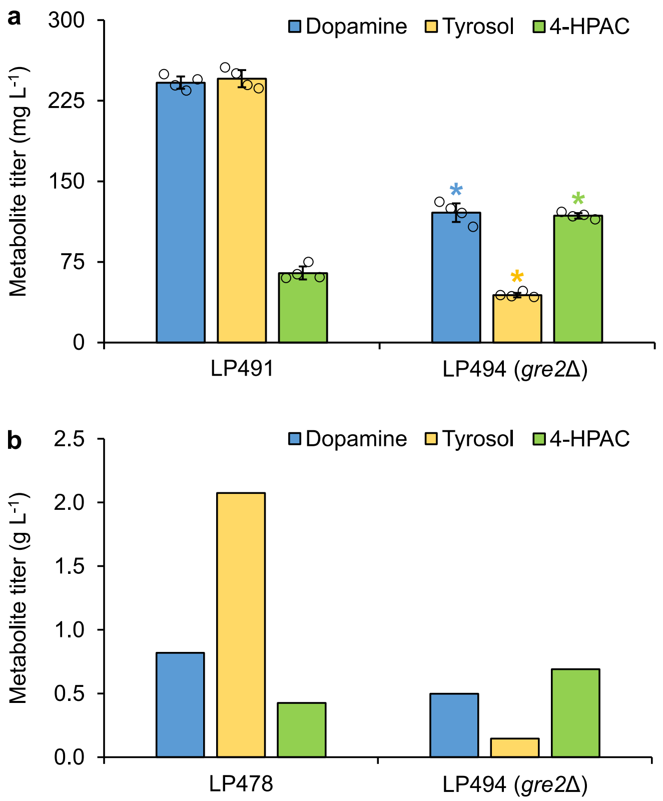

Supplementary Figure 10. Deletion of *GRE2* diminishes tyrosol synthesis in microtiter plate and pulsed fed-batch fermentor cultures. (**a**) Dopamine and fusel product synthesis in microtiter plate cultures. Strains LP491 and LP494 harbor deletions in five oxidoreductase genes (*ari1*Δ *adh6*Δ *ypr1*Δ *ydr541c*Δ *aad3*Δ), while LP494 contains an additional deletion in the *GRE2* gene, resulting in reduced levels of dopamine and tyrosol, and an increase in 4-HPAC concentration. Error bars represent the mean ± s.d. of n=4 independent biological samples. Asterisks (*) denote a significant increase or decrease ($P < 0.05$) in metabolite production relative to strain LP491. Statistical differences between control and derivative strains were tested using two-tailed Student's *t*-test. (**b**) Dopamine and fusel product synthesis in pulsed fed-batch fermentor cultures. Strain LP478 harbors deletions in five oxidoreductases (*ari1*Δ *adh6*Δ *ypr1*Δ *ydr541c*Δ *aad3*Δ), while LP494 contains an additional deletion in *GRE2*. Data is shown from the samples possessing the highest concentration of tyrosol from single fermentor experiments. Source data are provided in a Source Data file.

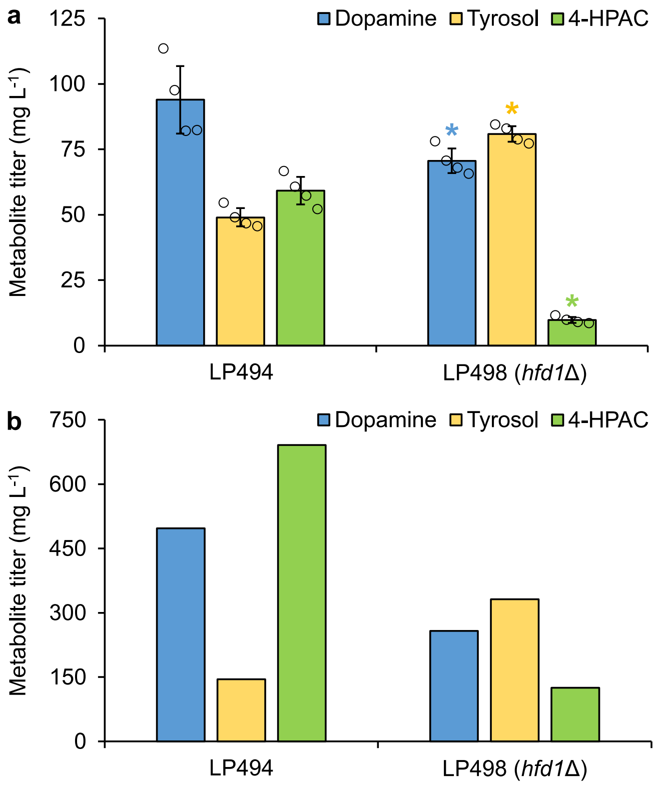

Supplementary Figure 11. Deletion of *HFD1* diminishes 4-HPAC synthesis in microtiter plate and pulsed fed-batch fermentor cultures. (**a**) Dopamine and fusel product synthesis in microtiter plate cultures. Strains LP494 and LP498 harbor deletions in six oxidoreductase genes (*ari1*Δ *adh6*Δ *ypr1*Δ *ydr541c*Δ *aad3*Δ *gre2*Δ), while LP498 contains an additional deletion in the *HFD1* aldehyde dehydrogenase gene. Deletion of *HFD1* results in reduced levels of dopamine and 4-HPAC. Error bars represent the mean ± s.d. of n=4 independent biological samples. Asterisks (*) denote a significant increase or decrease ($P < 0.05$) in metabolite production relative to strain LP494. Statistical differences between control and derivative strains were tested using two-tailed Student's *t*-test. (**b**) Dopamine and fusel product synthesis in pulsed fed-batch fermentor cultures. Data is shown from the samples possessing the peak concentration of 4-HPAC from single fermentor experiments. Source data are provided in a Source Data file.

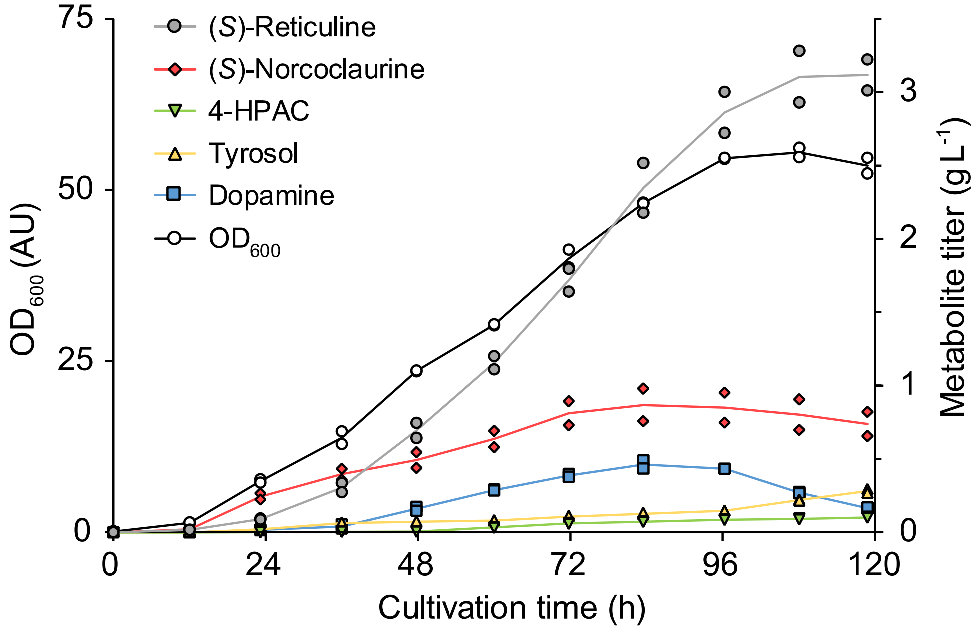

Supplementary Figure 12. Cultivation of an intermediate (*S*)-reticuline-producing strain (LP501) in a sucrose-pulsed fed-batch fermentor. Growth of biomass (OD_{600}) and accumulation of BIA metabolites in the culture medium during cultivation. Implementation of *gre2*Δ and *hfd1*Δ in an *ari1*Δ *adh6*Δ *ypr1*Δ *ydr541c*Δ *aad3*Δ background nearly abolishes fusel product synthesis under sucrose-pulsed fed-batch conditions. Data points from duplicate experiments are shown and the mean is depicted as a line. Fed-batch cultivation was performed as described in Supplementary Fig. 8. Source data are provided in a Source Data file.

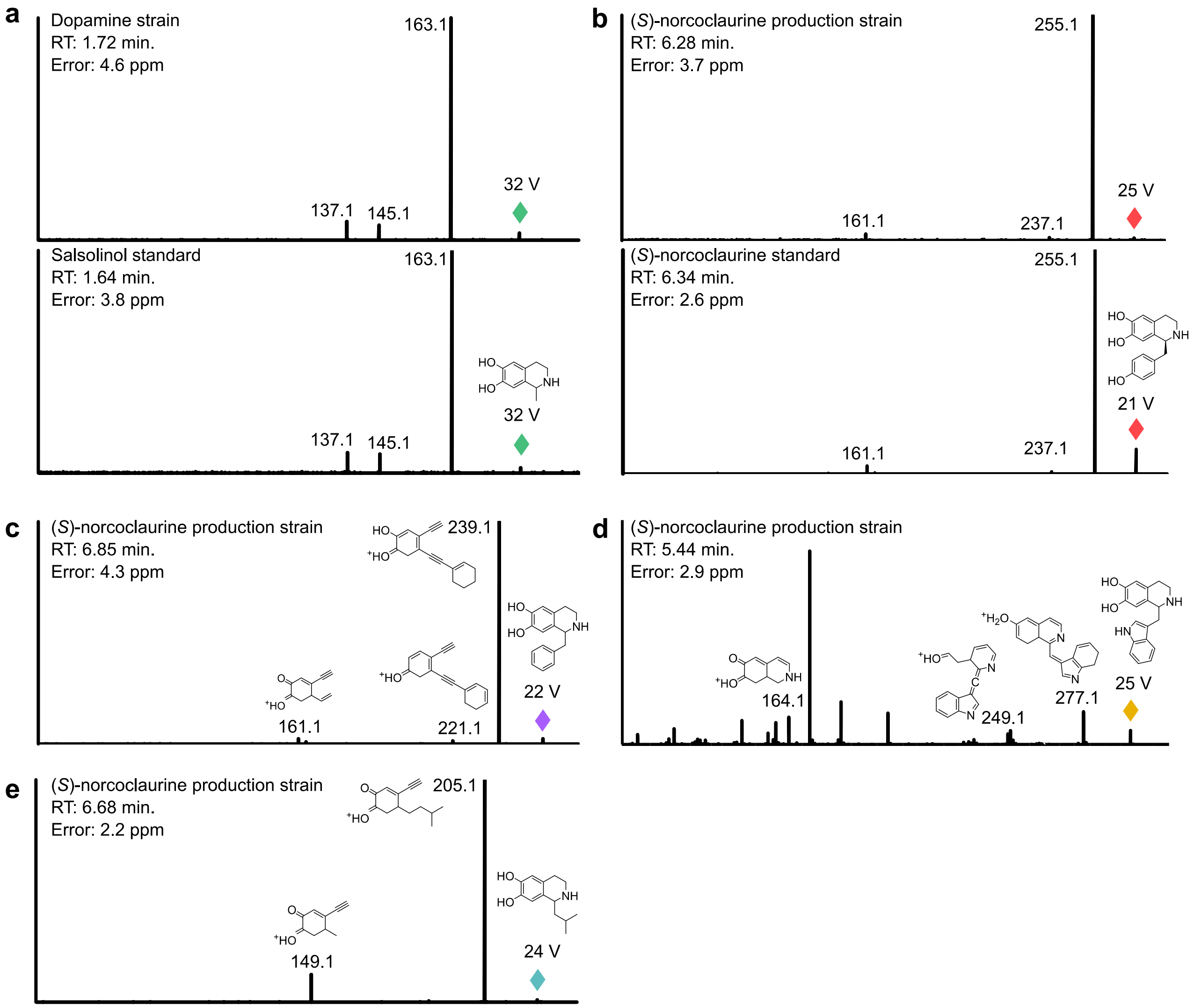

Supplementary Figure 13. Fragmentation spectra of substituted tetrahydroisoquinoline structures synthesized *de novo*. (**a**) Salsolinol (**13**). (**b**) (*S*)-Norcoclaurine (**3**). (**c**) Product **16**. (**d**) Product **19**. (**e**) Product **22**. Parent ions are depicted in color and collision energies (V) and mass errors (ppm) are shown. Fragmentation spectra of salsolinol (**13**) and (*S*)-norcoclaurine (**3**) were compared to spectra of authentic standards. Other structures were modelled using the CFM-ID tool[6]. Fragment structures and exact masses are shown for peaks that were matched using CFM-ID. Several observed peaks of **19** could not be matched with predicted fragments, as it has been reported that indole-containing molecules undergo complex rearrangements upon fragmentation[7]. Stereochemistry of non-canonical substituted tetrahydroisoquinolines is omitted. Salsolinol (**13**), (*S*)-norcoclaurine (**3**), **16**, and **22** were analyzed using FT-MS/MS. Product **19** was analyzed using QTOF-MS/MS. Repeating MS/MS fragmentations of all structures routinely yielded similar results.

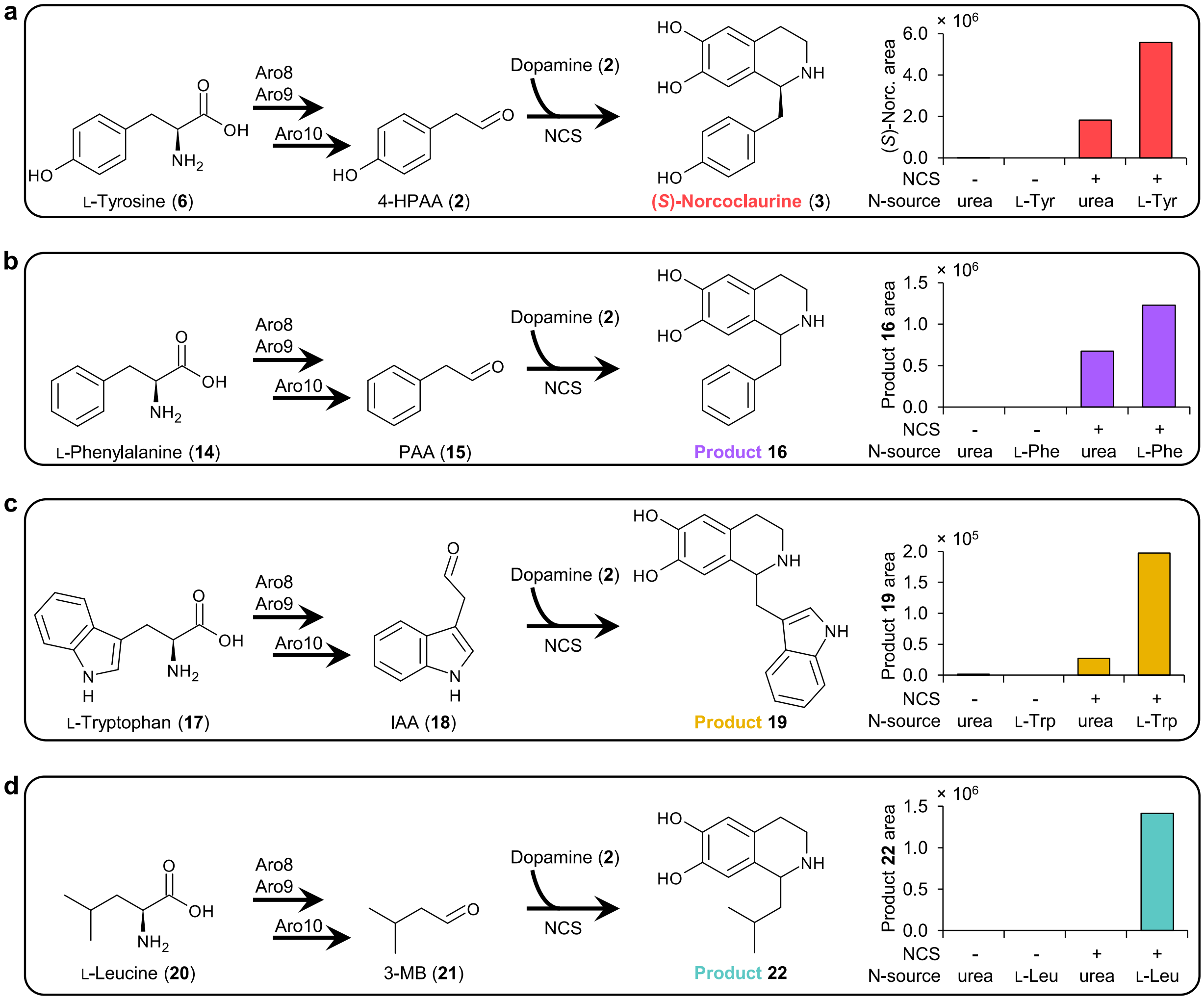

Supplementary Figure 14. Non-canonical substituted tetrahydroisoquinolines derive from amino acids. A dopamine-producing strain harboring *Cj*NCSΔN_{35} (strain LP385) was cultivated on urea or Ehrlich pathway amino acids as a sole source of nitrogen. (**a**) Synthesis of (*S*)-norcoclaurine (**3**) increases upon growth on L-tyrosine (**6**). (**b**) Synthesis of **16** increases upon growth on L-phenylalanine (**14**). (**c**) Synthesis of **19** increases upon growth on L-tryptophan (**17**). (**d**) Growth on L-leucine (**20**) is essential to observe formation of **22** using strain LP385. Stereochemistry of non-canonical substituted tetrahydroisoquinolines is omitted. Supplementation experiments were performed in duplicate and yielded similar results. Abbreviations: 4-HPAA, 4-hydroxyphenylacetaldehyde; IAA, indole acetaldehyde; 3-MB, 3-methylbutanal; PAA, phenylacetaldehyde; spont., spontaneous. Source data are provided in a Source Data file.

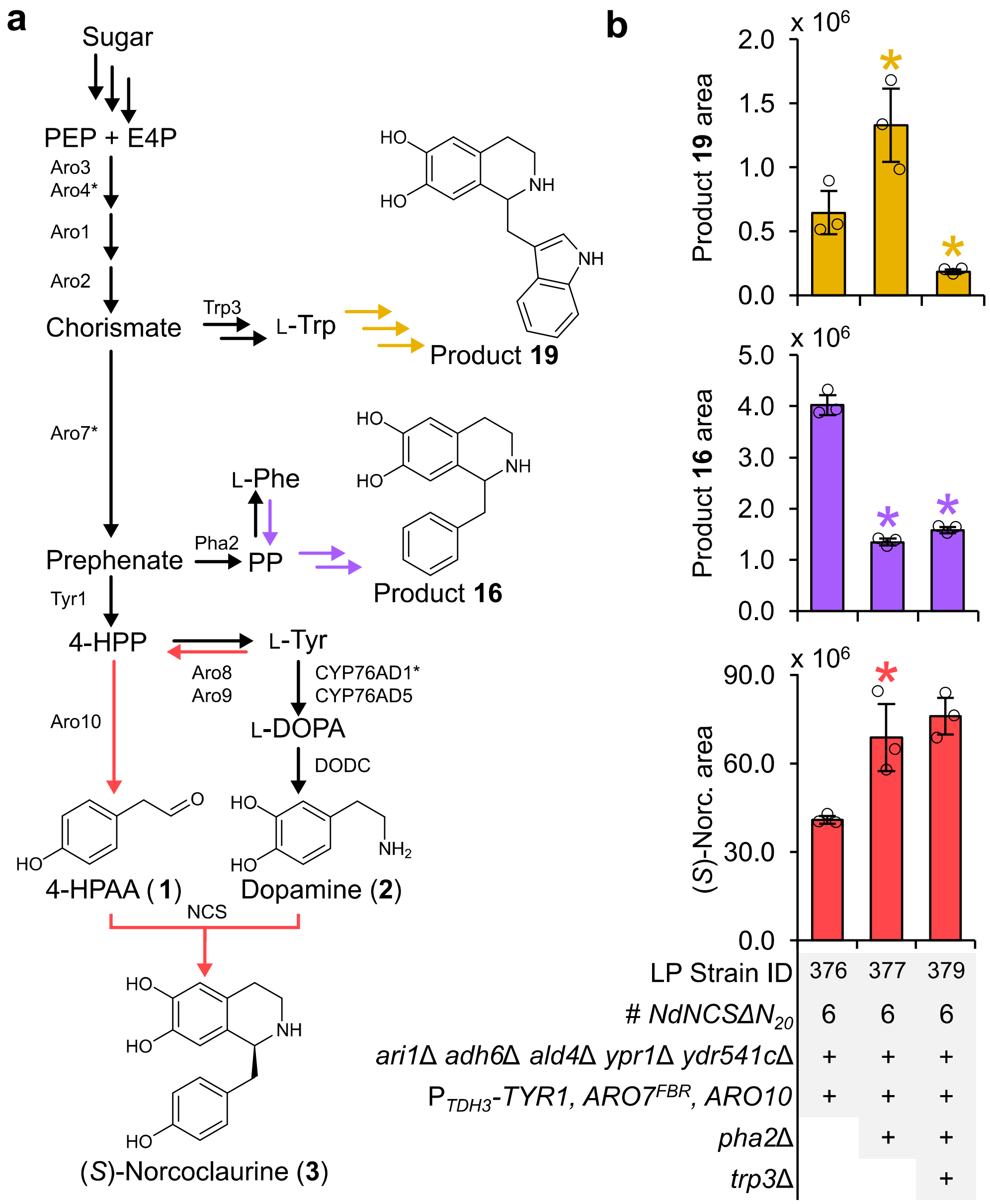

Supplementary Figure 15. Inactivation of *PHA2* and *TRP3* dramatically reduces levels of THIQ products 16 and 19, respectively. a, (*S*)-Norcoclaurine (**3**) biosynthesis pathway in engineered yeast showing pathways leading to products **16** and **19** from L-phenylalanine and L-tryptophan, respectively. Diverted Ehrlich pathways yielding THIQs from amino acids are shown in color. **b,** THIQ levels in culture supernatants of parent (LP376), *pha2*Δ (LP377), and *pha2*Δ *trp3*Δ (LP379) strains. Error bars represent the mean ± s.d. of n=3 independent biological samples. Asterisks (*) denote a significant increase or decrease ($P < 0.05$) in THIQ production relative to the precursor strain. Statistical differences between control and derivative strains were tested using two-tailed Student's *t*-test. Abbreviations: L-DOPA, L-3,4-dihydroxyphenylalanine; DODC, DOPA decarboxylase; E4P, erythrose-4-phosphate; 4-HPAA, 4-hydroxyphenylacetaldehyde; 4-HPAC, 4-hydroxyphenylacetic acid; NCS, norcoclaurine synthase; PEP, phosphoenolpyruvate; PP, phenylpyruvate; L-Phe, L-phenylalanine; L-Trp, L-tryptophan; L-Tyr, L-tyrosine. Source data are provided in a Source Data file.

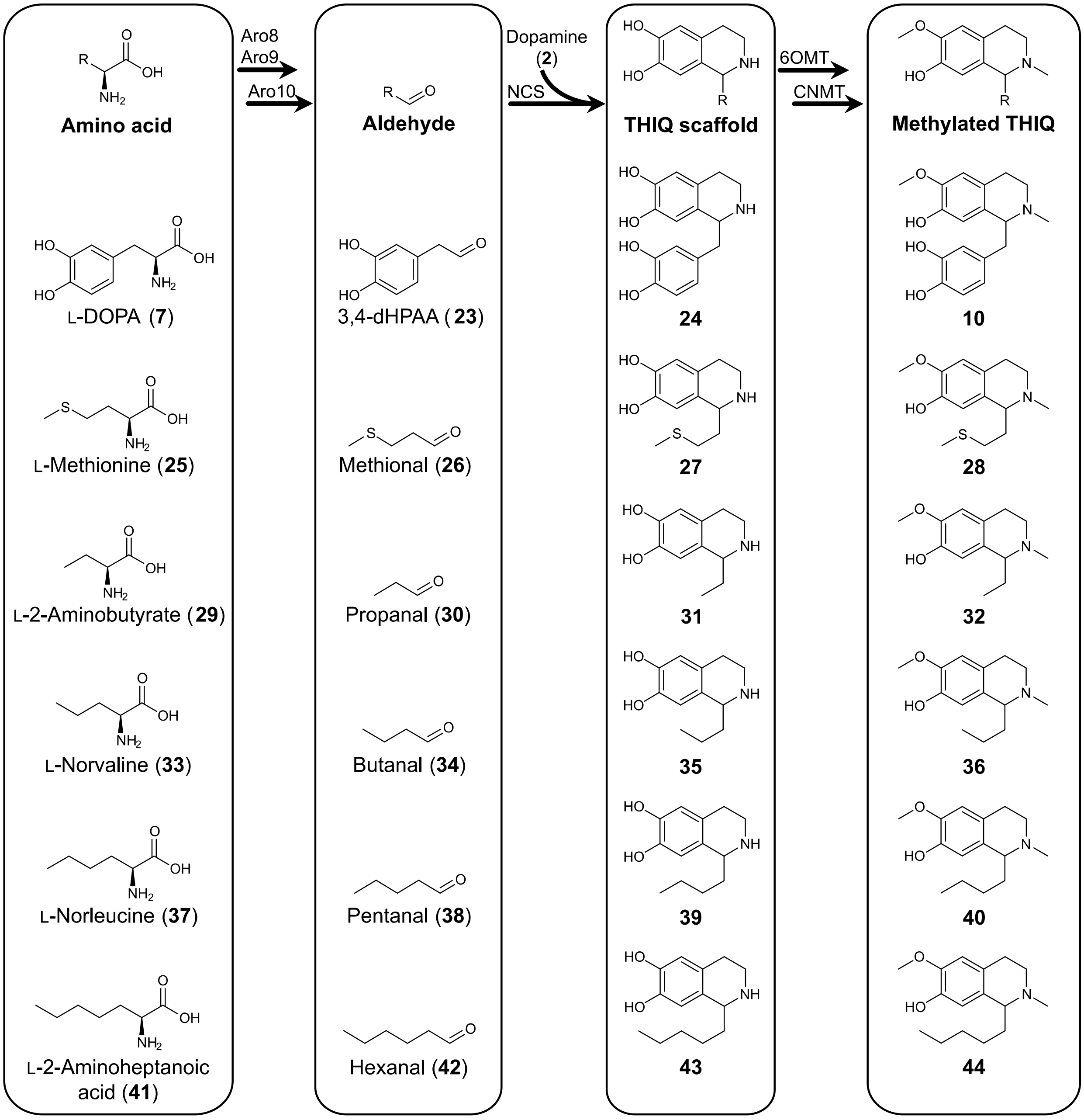

Supplementary Figure 16. Structures of substituted tetrahydroisoquinolines (THIQs) synthesized from supplemented amino acids. Amino acids are catabolized to the respective aldehyde species via the yeast Ehrlich pathway (Aro8/Aro9 + Aro10). In the presence of dopamine (**2**) and *Cj*NCSΔN_{35} (strain LP501), aldehydes are diverted to THIQ synthesis. Strain LP501 contains *Ps*6OMT and *Ps*CNMT methyltransferases for decorating THIQ scaffolds. Stereochemistry of substituted THIQs is omitted.

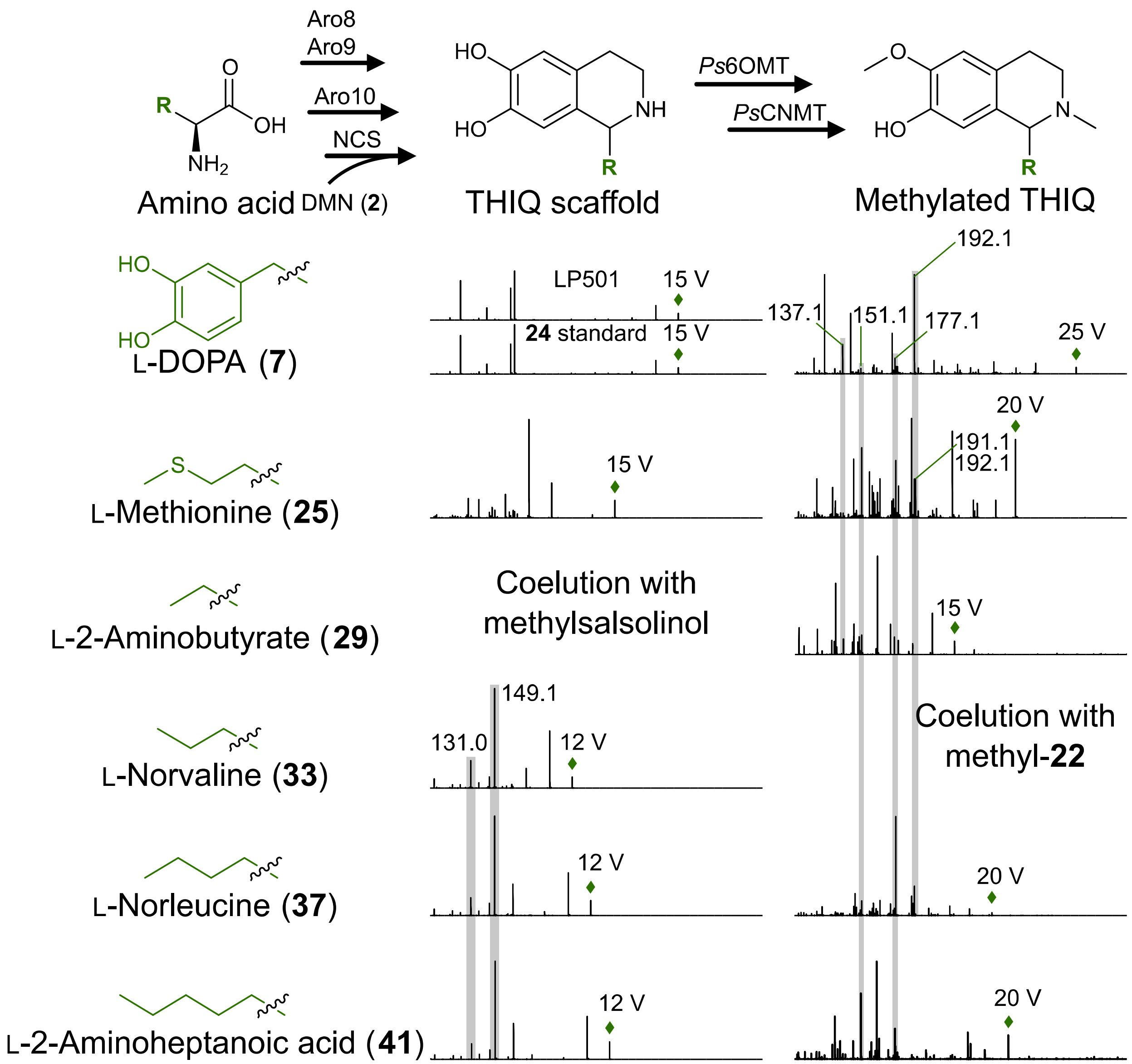

Supplementary Figure 17. Fragmentation spectra of substituted tetrahydroisoquinoline structures synthesized from supplemented amino acids. Parent ions are depicted with a green diamond. Fragmentation spectra of norlaudanosoline (**24**) was compared to that of authentic standard. Other structures were modelled using the CFM-ID tool[6]. Masses are shown for key peaks corresponding to THIQ fragments. Unmethylated alkyl-substituted tetrahydroisoquinolines yield characteristic fragments of *m/z* 131.0 and 149.1. Fragment ions of *m/z* 137.1, 151.1, 177.1, and 191.1 possess the 6OMT modification and *m/z* 192.1 possesses both 6OMT and CNMT modifications. Repeating MS/MS fragmentations routinely yielded similar results.

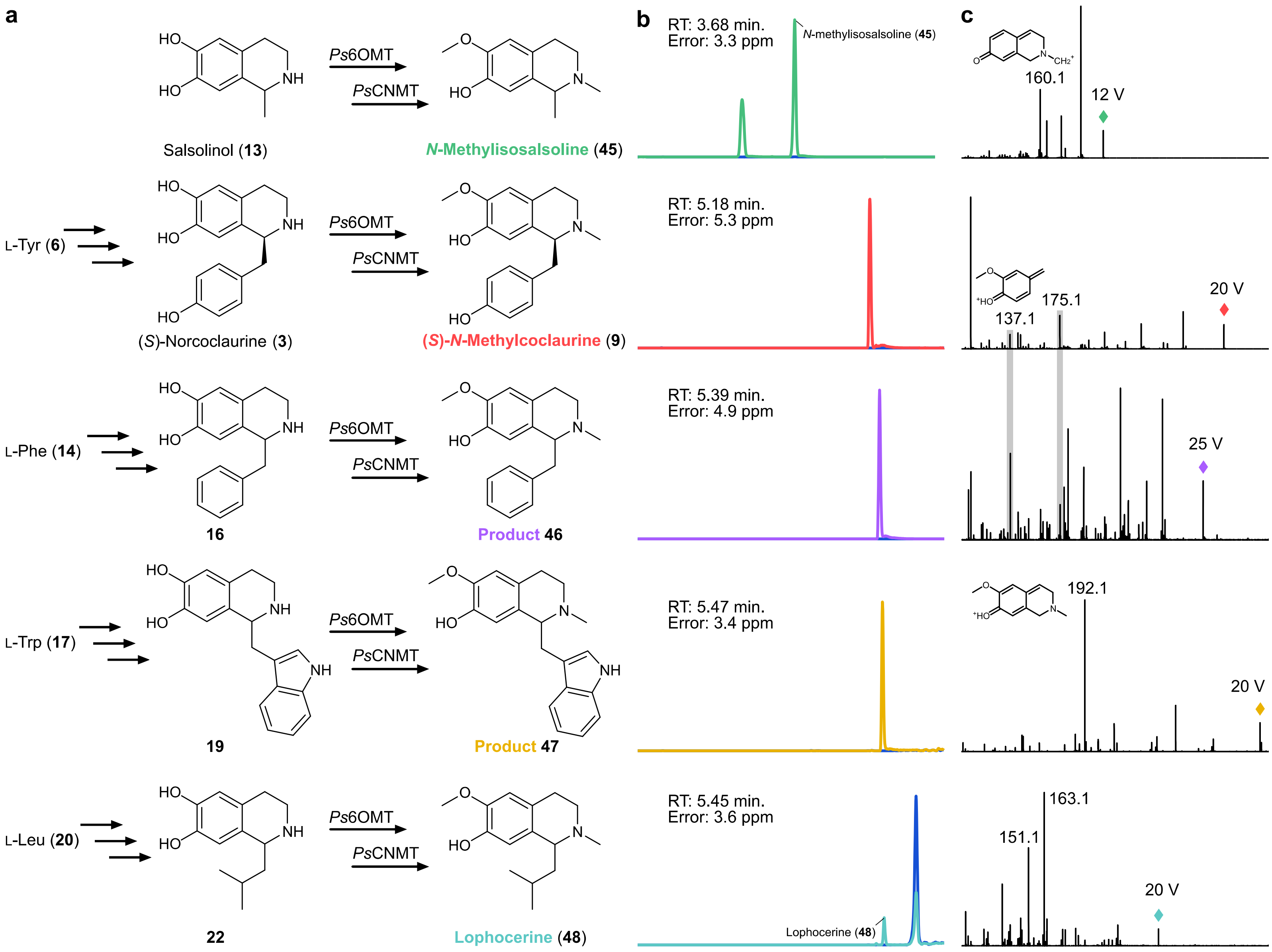
a
b
c
HO
NH
Salsolinol (13)
Ps6OMT
PsCNMT
N-Methylisosalsoline (45)
RT: 3.68 min.
Error: 3.3 ppm
N-methylisosalsoline (45)
160.1
12 V
L-Tyr (6)
(S)-Norcoclaurine (3)
(S)-N-Methylcoclaurine (9)
RT: 5.18 min.
Error: 5.3 ppm
137.1
175.1
20 V
L-Phe (14)
16
Product 46
RT: 5.39 min.
Error: 4.9 ppm
25 V
L-Trp (17)
19
Product 47
RT: 5.47 min.
Error: 3.4 ppm
192.1
20 V
L-Leu (20)
22
Lophocerine (48)
RT: 5.45 min.
Error: 3.6 ppm
Lophocerine (48)
163.1
151.1
20 V

Supplementary Figure 18. *De novo* synthesis of methylated substituted tetrahydroisoquinolines (THIQs) by an (*S*)-reticuline-producing host (strain LP501). (**a**) Decoration of *de novo* THIQ scaffolds by (*S*)-reticuline-pathway methyltransferases. Salsolinol (**13**), (*S*)-norcoclaurine (**3**), **16**, **19**, and **22** are synthesized from endogenous acetaldehyde (**12**), L-tyrosine (**6**), L-phenylalanine (**14**), L-tryptophan (**17**), and L-leucine (**20**), respectively. Strain LP501 produces *Ps*6OMT and *Ps*CNMT methyltransferases for decorating THIQ scaffolds, yielding *N*-methylisosalsoline (**45**), (*S*)-*N*-methylcoclaurine (**9**), **46**, **47**, and lophocerine (**48**). All of the depicted substituted THIQs and their methylated derivatives were synthesized *de novo* by all (*S*)-reticuline-producing strains. Stereochemistry of non-canonical substituted THIQs is omitted. (**b**) Ion-extracted LC-QTOF-MS chromatograms of strain LP501 grown on urea. Methylated substituted THIQs shifted in retention time (RT) relative to the canonical methylated product from L-tyrosine [(*S*)-*N*-methylcoclaurine (**9**)]. Growth of a dopamine-producing strain that lacks NCS, 6OMT, and CNMT enzymes (strain 1373) under the same conditions (blue) failed to generate peaks corresponding to substituted THIQs. All *m/z* values were calculated based on the expected theoretical structure of the respective compounds of interest and mass error (ppm) is shown. (**c**) QTOF-MS/MS fragmentation spectra of methylated substituted THIQ structures synthesized *de novo*. Parent ions are depicted using colored diamonds and collision energies are shown. Fragmentation spectra were modelled using the CFM-ID tool[6]. Structures and/or masses are shown for key peaks corresponding to methylated THIQ fragments. Fragments of *m/z* 137.1, 151.1, and 163.1 possess the 6OMT modification, *m/z* 160.1 contains the CNMT modification, and *m/z* 192.1 possesses both 6OMT and CNMT methyl groups. Fragments of *m/z* 137.1, 175.1, and 192.1 derive from fragmentation of (*S*)-reticuline[5]. Repeating MS/MS fragmentations of all structures routinely yielded similar results.

Supplementary Table 1. QTOF-MS analysis of substituted tetrahydroisoquinolines synthesized from supplemented amino acids.

THIQ product	THIQ formula	Retention time (min)	Expected THIQ mass (*m/z* + H^+)	Observed THIQ mass (*m/z* + H^+)	Mass error (ppm)
**24**	$C_{16}H_{17}NO_4$	3.17	288.1236	288.1230	2.1
**10**	$C_{18}H_{21}NO_4$	4.81	316.1549	316.1542	2.2
**27**	$C_{12}H_{17}NO_2S$	3.16	240.1058	240.1061	1.2
**28**	$C_{14}H_{21}NO_2S$	5.13	268.1371	268.1370	0.4
**31**	$C_{11}H_{15}NO_2$	1.98	194.1181	194.1185	2.1
**32**	$C_{13}H_{19}NO_2$	4.64	222.1494	222.1492	0.9
**35**	$C_{12}H_{17}NO_2$	2.91	208.1338	208.1332	2.9
**36**	$C_{14}H_{21}NO_2$	5.06	236.1650	236.1645	2.1
**39**	$C_{13}H_{19}NO_2$	4.63	222.1494	222.1499	2.2
**40**	$C_{15}H_{23}NO_2$	5.35	250.1807	250.1803	1.6
**43**	$C_{14}H_{21}NO_2$	5.34	236.1650	236.1645	2.1
**44**	$C_{16}H_{25}NO_2$	5.52	264.1963	264.1958	1.9

Supplementary Table 2. Plasmids utilized in this study.

Plasmid	Description	Source or reference
pCAS-G418	P_{RNR2}-*cas9*$_{NLS}$-T_{CYC1}, pUC, 2μ, P_{tRNA_Tyr}-3′HDV-gRNA-Scaffold-T_{SNR52}, P_{TEF1}-*kanMX*-T_{TEF1}	[8]
pBOT-His	CEN6/ARS4^{ori}, pMB1^{ori}, Amp^{R}, Kan^{R}, *HIS3*, P_{TEF1}-*GFP*^{S65T}-T_{PGI1}	[9]
pBOT-NdNCS	CEN6/ARS4^{ori}, pMB1^{ori}, Amp^{R}, Kan^{R}, *HIS3*, P_{TEF1}-*NdNCS*-T_{PGI1}	[9]
pBOT-ScNCS	CEN6/ARS4^{ori}, pMB1^{ori}, Amp^{R}, Kan^{R}, *HIS3*, P_{TEF1}-*ScNCS*-T_{PGI1}	[9]
pCAS-Hyg	P_{RNR2}-*cas9*$_{NLS}$-T_{CYC1}, pUC, 2μ, P_{tRNA_Tyr}-3′HDV-gRNA-Scaffold-T_{SNR52}, P_{TEF1}-*HphNTI*-T_{TEF1}	This study
pJET-LP5.T3	pMB1^{ori}, Amp^{R}, LP5.T3	This study
pBSC009	ColE1, Kan^{R}, *LEU2*, P_{TDH3}-*CjNCS*-T_{ENO2}	This study
pPSG325	ColE1, Kan^{R}, *LEU2*, P_{TDH3}-*CjNCSΔN*$_{20}$-T_{ENO2}	This study
pBSC011	ColE1, Kan^{R}, *LEU2*, P_{TDH3}-*CjNCSΔN*$_{35}$-T_{ENO2}	This study
pPSG834	CEN6/ARS4^{ori}, ColE1, Kan^{R}, *HIS3*, P_{CCW12}-*CYP76AD1*$^{W13L\ F309L}$-T_{ENO2}	This study
pPSG835	CEN6/ARS4^{ori}, ColE1, Kan^{R}, *HIS3*, P_{CCW12}-*CYP76AD5*-T_{ENO2}	This study
pPSG836	CEN6/ARS4^{ori}, ColE1, Kan^{R}, *HIS3*, P_{CCW12}-*CYP76AD6*-T_{ENO2}	This study
pPSG450	CEN6/ARS4^{ori}, ColE1, Kan^{R}, *HIS3*, P_{TEF1}-*EcNMCH*-T_{TDH3}, P_{TDH3}-*Ps6OMT*-T_{ADH1}, P_{PGK1}-*Ps4′OMT2*-T_{ENO1}, P_{TEF2}-*PsCNMT*-T_{SSA1}, P_{HHF1}-*AtATR2*-T_{ENO2},	This study

Supplementary Table 3. Expression cassettes utilized in this study.

Description	Cassette	Locus
Testing *Nd*NCS	FgF20-P_{TEF1}-*NdNCS*-T_{PGI1}-FgF20	FgF20
Testing *Sc*NCS	FgF20-P_{TEF1}-*ScNCS*-T_{PGI1}-FgF20	FgF20
Testing *Nd*NCSΔN_{20}	FgF20-P_{TEF1}-*NdNCSΔ*N_{20}-T_{PGI1}-FgF20	FgF20
Testing *Sc*NCSΔN_{20}	FgF20-P_{TEF1}-*ScNCSΔ*N_{20}-T_{PGI1}-FgF20	FgF20
Testing *Cj*NCS	FgF20-P_{TEF1}-*CjNCS*-T_{PGI1}-FgF20	FgF20
Testing *Cj*NCSΔN_{20}	FgF20-P_{TEF1}-*CjNCSΔ*N_{20}-T_{PGI1}-FgF20	FgF20
Testing *Cj*NCSΔN_{35}	FgF20-P_{TEF1}-*CjNCSΔ*N_{35}-T_{PGI1}-FgF20	FgF20
*Nd*NCS-GFP fusion	FgF20-P_{TEF1}-*NdNCS-GFP*-T_{PGI1}-FgF20	FgF20
*Sc*NCS-GFP fusion	FgF20-P_{TEF1}-*ScNCS-GFP*-T_{PGI1}-FgF20	FgF20
*Nd*NCSΔN_{20}-GFP fusion	FgF20-P_{TEF1}-*NdNCSΔ*N_{20}-*GFP*-T_{PGI1}-FgF20	FgF20
*Sc*NCSΔN_{20}-GFP fusion	FgF20-P_{TEF1}-*ScNCSΔ*N_{20}-*GFP*-T_{PGI1}-FgF20	FgF20
GFP control	FgF20-P_{TEF1}-*GFP*-T_{PGI1}-FgF20	FgF20
Expression of *noxE* in *adh1*Δ	FgF7-P_{TEF1}-*LlnoxE*-T_{IDP1}-FgF7	FgF7
Expression of *NdNCS* or *NdNCSΔ*N_{20}	FgF20-LV3-P_{TEF1}-*NdNCS*-T_{PGI1}-LV5-FgF20<br>FgF20-LV3-P_{TEF1}-*NdNCSΔ*N_{20}-T_{PGI1}-LV5-FgF20	FgF20
Overexpression of *TYR1*	USERXII-2-LV3-P_{TDH3}-*TYR1*-T_{TDH1}-LV5-USERXII-2	USERXII-2
Expression of *ARO7*^{FBR}	FgF16-LV3-P_{TDH3}-*ARO7*^{FBR}-T_{TDH1}-LV5-FgF16	FgF16
Overexpression of *ARO10*	FgF18-LV3-P_{TDH3}-*ARO10*-T_{TDH1}-LV5-FgF18	FgF18
Overexpression of *ARO2*	FgF19-LV3-P_{TDH3}-*ARO2*-T_{TDH1}-LV5-FgF19	FgF19
Expression of 1st copy of *CjNCSΔ*N_{35}	FgF24-LV3-P_{TDH3}-*CjNCSΔ*N_{35}-T_{TDH1}-LV5-FgF24	FgF24 (*PDC6*)
Reintroduction of *ALD4*	308a-LV3-P_{ALD4}-*ALD4*-T_{ALD4}-LV5-308a	308a
Expression of 2nd copy of *CjNCSΔ*N_{35}	USERXII-5-LV3-P_{TEF1}-*CjNCSΔ*N_{35}-T_{PGI1}-LV5-USERXII-5	USERXII-5
Expression of *CYP76AD5*	1309a-LV3-P_{TEF1}-*CYP76AD5*-T_{TDH1}-LV5-1309a	1309a
Introduction of reticuline biosynthesis pathway	106a-LV3-P_{TEF1}-*EcNMCH*-T_{TDH3}-P_{TDH3}-*Ps6OMT*-T_{ADH1}-P_{PGK1}-*Ps4′OMT2*-T_{ENO1}-P_{TEF2}-*PsCNMT*-T_{SSA1}-P_{HHF1}-*AtATR2*-T_{ENO2}-LV5-106a	106a
Expression of 2nd copy of *Ps4′OMT*	416d-LV3-P_{TDH3}-*Ps4′OMT2*-T_{TDH1}-LV5-416d	416d
Expression of 2nd copy of *Ps6OMT*	511b-LV3-P_{TDH3}-*Ps6OMT*-T_{TDH1}-LV5-511b	511b
Reintroduction of *PHA2* + *TRP3*	911b-LV3-P_{PHA2}-*PHA2*-T_{PHA2}-P_{TRP3}-*TRP3*-T_{TRP3}-LV5-911b	911b

Supplementary Table 4. ***S. cerevisiae*** **integration sites utilized in this study.**

**Target site ID**	**Target site sequence** [a]	**Reference**
FgF7	TATCCTGAATGTTCTCTCCCAGG	[9,10]
FgF16	TGTACCAAAAGTTATCCTGTAGG	[9,10]
FgF18	ATAGAATTACTATTGAAGAGTGG	[9,10]
FgF19	ATTCACTCTGCTAAGATTATCGG	[9,10]
FgF20	GTTAGAGCTGTTACAAGTTACGG	[9,10]
FgF24 (*PDC6*)	GTACAACGAAATCCAGACCTGGG	[9,10]
USERXII-1	GTCTTTGCCGGTTACCCATCTGG	[11]
USERXII-2	TCGAGAGAGTCGCCGATAGTAGG	[11]
USERXII-5	TTGTCACAGTGTCACATCAGCGG	[11]
106a	ATACGGTCAGGGTAGCGCCCTGG	[4]
308a	CACTTGTCAAACAGAATATAAGG	[4]
416d	TAGTGCACTTACCCCACGTTCGG	[4]
511b	CAGTGTATGCCAGTCAGCCACGG	[4]
911b	GTAATATTGTCTTGTTTCCCTGG	[4]
1309a	CCTGTGGTGACTACGTATCCAGG	[4]
AAD3	CAGGCGGAATGTAATAGGTGCGG	This study
AAD14	TCGTATTCAAGGAAACCGGGGGG	This study
ALD2+ALD3	GCTCAAGAATGTTCATATAAAGG	This study
ALD4	GGGTGTAGGTAAGCAGAATGAGG	This study
ALD5	TTAGAGTTTTTCGATGAGAATGG	This study
ALD6	ACACCGTTCGAGGTCAAGCCTGG	This study
ADH1	CTCTAATGAGCAACGGTATACGG	This study
ADH2	GCACTCTATTTATATGTGATAGG	This study
ADH3	AGCGAGTGTTCCTTTCTAAAAGG	This study
ADH4	CAATTGCTTTGTAGAGTTAACGG	This study
ADH5	TTACAATCTAGACAATACGAAGG	This study
ADH6	CACTTCACCTCGAGAACTGTTGG	This study
ADH7	AATCCCAATGTCATTTAATGCGG	This study
ARI1	AATTAGCATAGGATTTTCCGCGG	This study
GCY1	TAGGGAATTAAGGAGAGCAGCGG	This study
GRE2	TAGAATACGGAATTTTCTCGCGG	This study
HFD1	AGGCATATTGATTATCTAAAAGG	This study
NdNCS (N-terminus)	TTGGGTTGTGAAATTTCCCAAGG	This study
NdNCS (T_{PGI1}-LP5)	AGTTAGGTCTGGTATACTGGAGG	This study
PHA2	TCAGCGACAAAAGTAAACAGTGG	This study
SFA1	GTAATAATGGAATTTCATAGAGG	This study
T3	GCCAGTCAGAACACTAGAGGCGG	This study
TRP3	TCTATCGGGAATTACCACCAGGG	This study

YDR541C	GGCACCCAAAATAGATAGAGTGG	This study
YGL039W	GGGTTTGGCACAATTTGGCTTGG	This study
YPR1	GAAACCCAACACTGGAATGGAGG	This study

[a] PAMs are underlined.

Supplementary References

1 Heux, S., Cachon, R. & Dequin, S. Cofactor engineering in *Saccharomyces cerevisiae*: expression of a H_2O-forming NADH oxidase and impact on redox metabolism. *Metab. Eng.* **8**, 303-314 (2006).

2 Sunnadeniya, R. *et al.* Tyrosine hydroxylation in betalain pigment biosynthesis is performed by cytochrome P450 enzymes in beets (*Beta vulgaris*). *PLoS ONE* **11**, e0149417 (2016).

3 Lee, M. E., DeLoache, W. C., Cervantes, B. & Dueber, J. E. A highly characterized yeast toolkit for modular, multipart assembly. *ACS Synth Biol.* **4**, 975-986 (2015).

4 Reider Apel, A. *et al.* A Cas9-based toolkit to program gene expression in *Saccharomyces cerevisiae*. *Nucleic Acids Res.* **45**, 496-508 (2016).

5 DeLoache, W. C. *et al.* An enzyme-coupled biosensor enables (*S*)-reticuline production in yeast from glucose. *Nat. Chem. Biol.*, 465-471 (2015).

6 Allen, F., Pon, A., Wilson, M., Greiner, R. & Wishart, D. CFM-ID: a web server for annotation, spectrum prediction and metabolite identification from tandem mass spectra. *Nucleic Acids Res.* **42**, W94-W99 (2014).

7 Cao, S., Liu, H., Xu, W., Liao, X. & Zhao, Y. Characterizing the electrospray ionization mass spectral fragmentation pattern of indole derivatives synthesized from 2-keto glycosides. *J. Mass Spectrom.* **40**, 452-457 (2005).

8 Ryan, O. W. *et al.* Selection of chromosomal DNA libraries using a multiplex CRISPR system. *eLife* **3**, e03703 (2014).

9 Bourgeois, L., Pyne, M. E. & Martin, V. J. A highly characterized synthetic landing pad system for precise multicopy gene integration in yeast. *ACS Synth Biol.* **7**, 2675-2685 (2018).

10 Bai Flagfeldt, D., Siewers, V., Huang, L. & Nielsen, J. Characterization of chromosomal integration sites for heterologous gene expression in *Saccharomyces cerevisiae*. *Yeast* **26**, 545-551 (2009).

11 Mikkelsen, M. D. *et al.* Microbial production of indolylglucosinolate through engineering of a multi-gene pathway in a versatile yeast expression platform. *Metab. Eng.* **14**, 104-111 (2012).
