# Supplementary material for: A yeast platform for high-level synthesis of tetrahydroisoquinoline alkaloids
Source: Nat Commun. 2020 Jul 3;11:3337. doi: 10.1038/s41467-020-17172-x (PMC7335070; doi:10.1038/s41467-020-17172-x)
Supplement: Supplementary file 2 — Reporting Summary [file 41467_2020_17172_MOESM2_ESM.pdf]

nature research

Corresponding author(s): Vincent J.J. Martin

Last updated by author(s): Jun 14, 2020

Reporting Summary

Nature Research wishes to improve the reproducibility of the work that we publish. This form provides structure for consistency and transparency in reporting. For further information on Nature Research policies, see Authors & Referees and the Editorial Policy Checklist .

Statistics

For all statistical analyses, confirm that the following items are present in the figure legend, table legend, main text, or Methods section.

n/a	Confirmed	
☐	☒	The exact sample size (*n*) for each experimental group/condition, given as a discrete number and unit of measurement
☐	☒	A statement on whether measurements were taken from distinct samples or whether the same sample was measured repeatedly
☐	☒	The statistical test(s) used AND whether they are one- or two-sided<br>*Only common tests should be described solely by name; describe more complex techniques in the Methods section.*
☒	☐	A description of all covariates tested
☒	☐	A description of any assumptions or corrections, such as tests of normality and adjustment for multiple comparisons
☐	☒	A full description of the statistical parameters including central tendency (e.g. means) or other basic estimates (e.g. regression coefficient) AND variation (e.g. standard deviation) or associated estimates of uncertainty (e.g. confidence intervals)
☐	☒	For null hypothesis testing, the test statistic (e.g. F, t, r) with confidence intervals, effect sizes, degrees of freedom and P value noted<br>*Give P values as exact values whenever suitable.*
☒	☐	For Bayesian analysis, information on the choice of priors and Markov chain Monte Carlo settings
☒	☐	For hierarchical and complex designs, identification of the appropriate level for tests and full reporting of outcomes
☒	☐	Estimates of effect sizes (e.g. Cohen's *d*, Pearson's *r*), indicating how they were calculated

Our web collection on statistics for biologists contains articles on many of the points above.

Software and code

Policy information about availability of computer code

Data collection	Agilent ChemStation for HPLC-MS data<br>Applikon BioXpert for bioreactor data
Data analysis	Agilent MassHunter for Q-TOF-MS analysis<br>Thermo Xcalibur for FT-MS analysis<br>Fiji for microscopy analysis<br>Microsoft Excel and GraphPad for analyzing metabolite data

For manuscripts utilizing custom algorithms or software that are central to the research but not yet described in published literature, software must be made available to editors/reviewers. We strongly encourage code deposition in a community repository (e.g. GitHub). See the Nature Research guidelines for submitting code & software for further information.

Data

Policy information about availability of data

All manuscripts must include a data availability statement. This statement should provide the following information, where applicable:

- Accession codes, unique identifiers, or web links for publicly available datasets
- A list of figures that have associated raw data
- A description of any restrictions on data availability

The source data underlying the findings of this work are provided as a Source Data file.

Field-specific reporting

Please select the one below that is the best fit for your research. If you are not sure, read the appropriate sections before making your selection.

- [x] Life sciences
- [ ] Behavioural & social sciences
- [ ] Ecological, evolutionary & environmental sciences

For a reference copy of the document with all sections, see nature.com/documents/nr-reporting-summary-flat.pdf

Life sciences study design

All studies must disclose on these points even when the disclosure is negative.

Sample size	Unless stated otherwise, all quantitative experiments were performed with at least three biological replicates, which is the preferred approach in this field. The fed-batch bioreactor experiment reported in the main text (Fig. 2c), as well as Figure S12, were performed in duplicate, while the bioreactor data depicted in Fig. S8a, S10b, and S11b were attained from single runs. Qualitative experiments involving non-numerical outcomes, such as the presence of LC-MS peaks or MS-MS spectra, were performed using at least two replicates although only one outcome is depicted for clarity (Fig. 4, S13, S14, S17, S18).
Data exclusions	No relevant data was excluded.
Replication	All attempts for replication were successful. Two to four biological replicates were included for all quantitative measurements. All qualitative experiments were replicated at least once and produced comparable outcomes in all instances.
Randomization	All strains were characterized from randomly picked colonies on agar plates in triplicate.
Blinding	Not applicable. All samples within an experiment were processed identically in parallel using standard procedures and protocols that should not bias outcomes.

Reporting for specific materials, systems and methods

We require information from authors about some types of materials, experimental systems and methods used in many studies. Here, indicate whether each material, system or method listed is relevant to your study. If you are not sure if a list item applies to your research, read the appropriate section before selecting a response.

Materials & experimental systems

n/a	Involved in the study
[x]	[ ] Antibodies
[x]	[ ] Eukaryotic cell lines
[x]	[ ] Palaeontology
[x]	[ ] Animals and other organisms
[x]	[ ] Human research participants
[x]	[ ] Clinical data

Methods

n/a	Involved in the study
[x]	[ ] ChIP-seq
[x]	[ ] Flow cytometry
[x]	[ ] MRI-based neuroimaging
