# Supplementary material for: A yeast platform for high-level synthesis of tetrahydroisoquinoline alkaloids
Source: Nat Commun. 2020 Jul 3;11:3337. doi: 10.1038/s41467-020-17172-x (PMC7335070; doi:10.1038/s41467-020-17172-x)
Supplement: Supplementary file 3 — Description of Additional Supplementary Files [file 41467_2020_17172_MOESM3_ESM.docx]

**Description of Additional Supplementary Files**

File name: Supplementary Data 1
Description: Putative substituted tetrahydroisoquinoline products queried using FT-MS.

^a^ Endogenous carbonyl species were compiled from previous studies of *S. cerevisiae* metabolism[^1^](#_ENREF_1)^,^[^2^](#_ENREF_2)

^b^ The product of dopamine+2MB is isomeric with that of dopamine+3MB (*m/z* + H^+^ = 222.1494). The identity of the putative THIQ product was deduced to be dopamine+3MB since growth on l-leucine, rather than l-isoleucine, generates the corresponding LC-MS peak (refer to Supplementary Fig. 14). Further, α-substituted carbonyls, such as 2MB, are not well tolerated by NCS[^3^](#_ENREF_3).

File name: Supplementary Data 2
Description: Oligonucleotides utilized in this study.

File name: Supplementary Data 3
Description: Strains utilized in this study.

^a^ Intermediate strains used in the construction of the final (*S*)-reticuline production host (LP507) are shaded.

File name: Supplementary Data 4
Description: Genes and synthetic DNAs synthesized in this study.

^a^ NCS regions corresponding to ΔN_20_ truncations are bolded and underlined and regions corresponding to ΔN_35_ truncations are bolded. The synthetic T3 Cas9 target site is underlined in LP5.T3.

References

1 Alves, Z. *et al.* Exploring the *Saccharomyces cerevisiae* volatile metabolome: indigenous versus commercial strains. *PLoS One* **10**, e0143641 (2015).

2 Hazelwood, L. A., Daran, J.-M., van Maris, A. J., Pronk, J. T. & Dickinson, J. R. The Ehrlich pathway for fusel alcohol production: a century of research on *Saccharomyces cerevisiae* metabolism. *Appl. Environ. Microbiol.* **74**, 2259-2266 (2008).

3 Roddan, R. *et al.* The acceptance and kinetic resolution of alpha-methyl substituted aldehydes by norcoclaurine synthases. *ACS Catalysis* (2019).
